# Predictors of in-hospital COVID-19 mortality: A comprehensive systematic review and meta-analysis exploring differences by age, sex and health conditions

**DOI:** 10.1371/journal.pone.0241742

**Published:** 2020-11-03

**Authors:** Arthur Eumann Mesas, Iván Cavero-Redondo, Celia Álvarez-Bueno, Marcos Aparecido Sarriá Cabrera, Selma Maffei de Andrade, Irene Sequí-Dominguez, Vicente Martínez-Vizcaíno

**Affiliations:** 1 Health and Social Research Centre, Universidad de Castilla-La Mancha, Cuenca, Spain; 2 Postgraduate Program in Public Health, Universidade Estadual de Londrina, Londrina, Paraná, Brasil; 3 Universidad Politécnica y Artística del Paraguay, Asunción, Paraguay; 4 Facultad de Ciencias de la Salud, Universidad Autónoma de Chile, Talca, Chile; Institute of Tropical Medicine Antwerp, BELGIUM

## Abstract

**Objective:**

Risk factors for in-hospital mortality in confirmed COVID-19 patients have been summarized in numerous meta-analyses, but it is still unclear whether they vary according to the age, sex and health conditions of the studied populations. This study explored these variables as potential mortality predictors.

**Methods:**

A systematic review was conducted by searching the MEDLINE, Scopus, and Web of Science databases of studies available through July 27, 2020. The pooled risk was estimated with the odds ratio (p-OR) or effect size (p-ES) obtained through random-effects meta-analyses. Subgroup analyses and meta-regression were applied to explore differences by age, sex and health conditions. The MOOSE guidelines were strictly followed.

**Results:**

The meta-analysis included 60 studies, with a total of 51,225 patients (12,458 [24.3%] deaths) from hospitals in 13 countries. A higher in-hospital mortality risk was found for dyspnoea (p-OR = 2.5), smoking (p-OR = 1.6) and several comorbidities (p-OR range: 1.8 to 4.7) and laboratory parameters (p-ES range: 0.3 to -2.6). Age was the main source of heterogeneity, followed by sex and health condition. The following predictors were more markedly associated with mortality in studies with patients with a mean age ≤60 years: dyspnoea (p-OR = 4.3), smoking (p-OR = 2.8), kidney disease (p-OR = 3.8), hypertension (p-OR = 3.7), malignancy (p-OR = 3.7), diabetes (p-OR = 3.2), pulmonary disease (p-OR = 3.1), decreased platelet count (p-ES = -1.7), decreased haemoglobin concentration (p-ES = -0.6), increased creatinine (p-ES = 2.4), increased interleukin-6 (p-ES = 2.4) and increased cardiac troponin I (p-ES = 0.7). On the other hand, in addition to comorbidities, the most important mortality predictors in studies with older patients were albumin (p-ES = -3.1), total bilirubin (p-ES = 0.7), AST (p-ES = 1.8), ALT (p-ES = 0.4), urea nitrogen (p-ES), C-reactive protein (p-ES = 2.7), LDH (p-ES = 2.4) and ferritin (p-ES = 1.7). Obesity was associated with increased mortality only in studies with fewer chronic or critical patients (p-OR = 1.8).

**Conclusion:**

The prognostic effect of clinical conditions on COVID-19 mortality vary substantially according to the mean age of patients.

**PROSPERO registration number:**

CRD42020176595.

## Introduction

The rapid global spread of COVID-19 beginning in December 2019 [1] and the countless associated health, social and economic impacts have resulted in an exponential increase in scientific publications from independent groups and global coalitions [2]. In addition to aspects related to the spread of the disease, the profile and symptoms of patients [3, 4] and the drugs being developed to prevent and treat it [5], much interest has been devoted to potential predictors of hospital mortality from COVID-19. The identification of clinical aspects observed at hospital admission among patients with a worse prognosis has been the focus of many meta-analyses. Although the accumulated evidence consistently indicates that worse prognosis is related to old age [6], the male sex [7] and the presence of comorbidities [8–10], it is still unclear whether other potential predictors of mortality, such as smoking, clinical symptoms or laboratory parameters, vary between populations with different sociodemographic and epidemiological profiles.

To the best of our knowledge, no meta-analysis specifically designed to assess the role of age, sex and health condition of the studied populations with a comprehensive list of potential clinical predictors of mortality in hospitalized patients with confirmed COVID-19 has been reported. Thus far, Zhang et al. [11] observed that the association between hypertension and mortality was stronger in studies with patients who are less than 50 years old (OR = 6.4) than in those 50 years old and older (OR = 2.6), although the authors included only six studies in their meta-analysis. In another study, Pranata et al. [12] used meta-regression methods and found an association between hypertension and increased composite worse outcome, including mortality, which is influenced by sex (more strongly when the population is less than 55% male patients), but not age. While the call for epidemiological data to be presented by age and sex groups has not been met [13], alternative proposals are requiered to provide a deeper understanding of how mortality risk factors vary according to the sociodemographic and epidemiological profiles of patients.

Additionally, with the extraordinary speed and quantity of publications on COVID-19 mortality predictors that have emerged in recent months, some meta-analyses have included studies with overlapping data from patients from the same hospitals [10, 14, 15] and preprint studies [8, 16, 17]. Although such methodological aspects are partially justified limitations considering the urgency for evidence, it is important to make efforts to overcome these limitations to yield results with greater precision and reliability.

Therefore, this systematic review and meta-analysis adds to the available evidence by synthesizing the results of primary studies and providing pooled effect size estimators for a comprehensive set of potential predictors of in-hospital mortality in patients with confirmed COVID-19. Concretely, we estimated the potential risk associated with symptoms and pre-existing chronic conditions and comorbidities reported on admission, and with specific laboratory parameters such as routine blood tests, coagulation, liver and kidney function and inflammatory factors. Subgroup analyses and meta-regression explored whether these factors vary according to age, sex and baseline health conditions of the studied populations.

## Methods

This systematic review with meta-analyses is reported according to the MOOSE guidelines [18] followed the recommendations of the Cochrane Collaboration Handbook [19] and was registered in PROSPERO (registration number: CRD42020176595). No ethics approval was required given the nature of the study.

### Search strategy and study selection

We systematically searched the MEDLINE (via PubMed), Scopus and Web of Science databases from December 2019 to July 27, 2020. The search was specifically focused on peer-reviewed studies that analysed demographic characteristics, clinical status, pre-existing comorbidities, and laboratory parameters on hospital admission as potential risk factors for higher mortality in subjects with confirmed COVID-19. The full search strategy is presented in the (**S1 Appendix**). The literature search was complemented by reviewing the citations of the articles considered eligible for the systematic review. No language restriction was applied.

The criteria for the inclusion of studies were as follows: (i) participants—100 and more patients with confirmed COVID-19; (ii) design—observational studies (prospective or retrospective) with primary individual data for each mortality outcome group, i.e., nonsurvivors and survivors; (iii) exposure variables—sociodemographic characteristics, clinical symptoms and signs, pre-existing comorbidities and the laboratory parameters routine blood tests, coagulation indicators, liver and kidney function and inflammatory factors; and (iv) outcome—all patients followed up to definitive hospital discharge or COVID-19 mortality.

The criteria for the exclusion of studies were as follows: (i) noneligible publication types, such as case series, family case studies or editorials and letters to the editor; (ii) studies with no deaths registered; and (iii) studies including only patients with specific diseases (cancer, organ transplantation, etc.) or from specific occupations or conditions (health professionals, prisoners, etc.). The literature search and the selection of the studies were independently conducted by two reviewers (AM and IC-R), and disagreements were solved by consensus or through the involvement of a third researcher (CA-B).

### Data extraction and risk of bias assessment

The following data were extracted from the original reports: (1) date of publication; (2) clinical setting; (3) timing of subjects’ admission to the hospital; (4) sample characteristics (sample size, percentage of nonsurvivors, percentage of patients followed-up until discharge or death, percentage of men, mean or median age).

Data extraction and quality assessment were independently performed by two reviewers (AM and CA-B), and inconsistencies were solved by consensus or by involving a third researcher (IC-R). When there were different units of measurement for the same parameter, we converted them to enable the meta-analyses.

The Quality In Prognosis Studies (QUIPS) [20] was used to assess the risk of bias and quality of the included studies, as presented in the (**S2 Table**).

### Statistical analyses and data synthesis

The odds ratio (OR) and its respective 95% confidence interval (CI) were calculated for nonsurvivors and survivors among exposed and nonexposed individuals for each potential predictor. Regarding the laboratory indicators, we calculated the following for each parameter: i) the pooled mean estimate for nonsurvivors and survivors, ii) the pooled mean difference (MD) between nonsurvivors and survivors, and iii) the effect size (ES) statistic, using Cohen’s d index [21]. These mean values and mean differences were calculated and presented in the unit of measurement most frequently adopted for each laboratory parameter. Values in different units of measure were converted to the reference unit when necessary.

The DerSimonian and Laird method [22] was used to compute the pooled mean estimates, MD, ES and the OR and its respective 95% CI. Meta-analyses with random-effects models were performed for each potential predictor. The heterogeneity of the results across studies was assessed using the I^2^ statistic [23]. We selected a random effect model because moderate or high heterogeneity (I^2^ >50%) was observed in 38 of 41 predictors under analysis. Because some studies conducted in China obtained data from the same hospital and time interval, we only considered the study with later end date when analysing each predictor to avoid overrepresentation of data from the same patients [24]. We provided a list of those studies and the order of preference considered for analysis in the (**S2 Appendix**).

Subgroups meta-analyses were applied when the percentage of total variation across studies due to heterogeneity was moderate or high (I^2^ >50%) [23]. The pooled OR (p-OR) and ES (p-ES) of each potential predictor was recalculated in subgroups of studies defined according the proportion of patients who were elderly, men and in poor health condition. For this purpose, we considered two groups of predominant age (older adults: the mean age of patients was >60 years; younger adults: ≤60 years) [6], two groups of predominant sex (≥60% of male patients; <60%) and two groups of predominant health condition (poor: all patients were in critical or severe condition or the most prevalent chronic condition was >50%; regular: studies that did not fit the poor criteria). The subgroup classification of the included studies and the corresponding values considered are available in the (**S1 Table**).

For the purpose of meta-regression, the continuous mean age (years), as well as the proportion of male sex and of the highest prevalence of chronic condition or critical status were considered as continuous adjustment variables. Thus, three models adjusted the pooled-OR for age, sex and health condition for each predictor and the percentage of residual variation due to heterogeneity was generated [25].

Sensitivity analyses were conducted to assess the robustness of the summary estimates and to determine whether any particular study accounted for a large proportion of the heterogeneity. Additionally, post-hoc sensitivity analyses were performed by removing studies with a moderate risk of bias in more than one domain of the QUIPS tool [20].

Finally, publication bias was evaluated by visual inspection of funnel plots and by using the method proposed by Egger, considering a p-value of <0.10 to be statistically significant [26].

Statistical analyses were performed using the commands metan and metareg of STATA SE software, version 15 (StataCorp, College Station, TX, USA). All analyses were performed by two reviewers (AM and IC-R).

## Results

**Fig 1:**
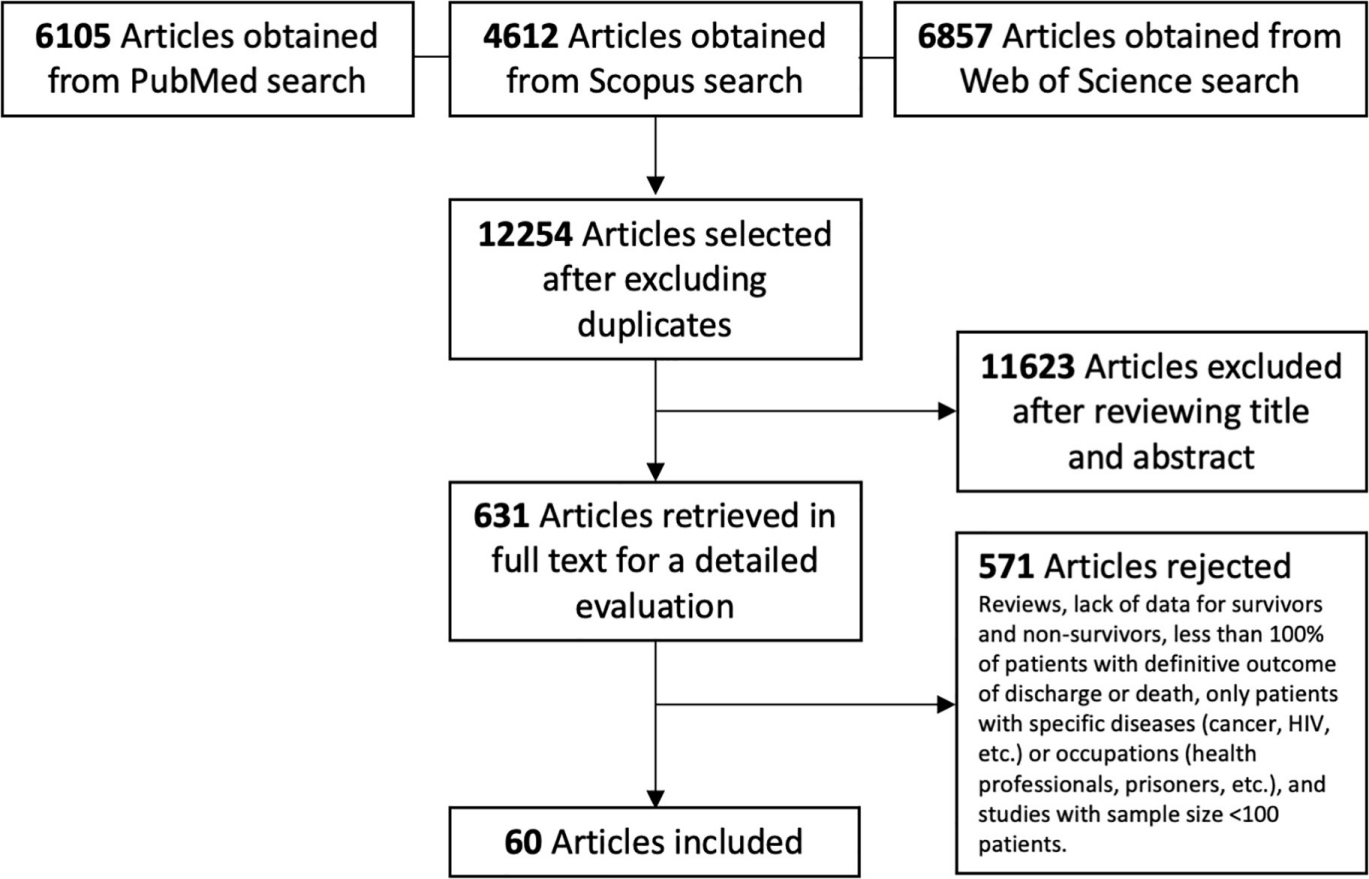
Flow diagram of the literature search and studies selection.

We identified 60 studies [27–86] that met the inclusion criteria (**Fig 1**). The studies were conducted in hospitals from 13 countries. The timing of hospital admission of subjects ranged from December 24, 2019 and May 17, 2020. The mean age of the populations included ranged between 40 and 73 years, with sample sizes ranging from 100 to 7,371 participants. In total, 51,225 patients were included, 12,458 of whom (24.3%) were nonsurvivors (**Table 1**).

**Table 1:** Characteristics of the included studies.

Authors	Country	Patient’s admission		Patients, n	Deaths, n (%)	Male sex, %	Age^a^ (years)
		From	To				
Aloisio E et al. [27]	Italy	Feb 21, 2020	Mar 31, 2020	427	89 (20.8)	68.60	61.3 ± 6.6
Amit M et al. [28]	Israel	Mar 5, 2020	Apr 27, 2020	156	87 (55.8)	69.00	71.5 ± 6.3
Asghar MS et al. [29]	Pakistan	Mar, 2020	Apr, 2020	100	22 (22.0)	69.00	52.6 ± 15.7
Baqui P et al. [30]	Brazil (Central-South)	Feb 27, 2020	May 4, 2020	6021	2457 (40.8)	58.00	58.2 ± 17.8
	Brazil (North)	Feb 27, 2020	May 4, 2020	1350	871 (64.5)	58.90	58.8 ± 19.4
Bonetti G et al. [31]	Italy	Mar 1, 2020	Mar 30, 2020	144	70 (48.6)	66.70	69.1 ± 8.9
Borghesi A et al. [32]	Italy	Mar 4, 2020	Mar 24, 2020	302	65 (21.5)	64.20	67.0 ± 5.8
Borobia AM et al. [33]	Spain	Feb 25, 2020	Apr 19, 2020	2226	460 (20.7)	48.20	61.5 ± 9.2
Brill SE et al. [34]	UK	Mar 10, 2020	Apr 8, 2020	410	173 (42.2)	59.8	69.9 ± 7.0
Cao J et al. [35]	China	Jan 3, 2020	Feb 1, 2020	102	17 (16.7)	52.00	53.0 ± 8.7
Carter B et al. [36]	UK/Italy	Feb 27, 2020	Apr 28, 2020	1564	425 (27.2)	57.70	73.0 ± 6.3
Chen F et al. [37]	China	Jan 1, 2020	Feb 15, 2020	660	82 (12.4)	44.70	53.0 ± 9.8
Chen R et al. [38]	China	NA	Mar 22, 2020	548	103 (18.8)	57.10	56.0 ± 14.5
Chen T et al. [39]	China	Jan 13, 2020	Feb 12, 2020	274	113 (41.2)	62.40	59.5 ± 7.5
Cheng A et al. [40]	China	Feb 8, 2020	Mar 11, 2020	305	85 (27.9)	60.00	63.3 ± 5.5
Ciceri F et al. [41]	Italy	Feb 25, 2020	Mar 24, 2020	386	95 (24.6)	71.80	65.7 ± 7.4
Deng Y et al. [42]	China	Jan 1, 2020	Feb 21, 2020	225	109 (48.4)	55.10	55.1 ± 14.1
Du RH et al. [43]	China	Dec 25, 2019	Feb 7, 2020	179	21 (11.7)	54.20	57.6 ± 13.7
Gao S et al. [44]	China	Jan 23, 2020	Feb 29, 2020	210	35 (16.7)	48.00	71.5 ± 2.9
Garcia PDW et al. [45]	Switzerland, Spain, Italy, France, Germany	NA	Apr 22, 2020	639	97 (15.2)	75.10	62.5 ± 5.2
Gavin W et al. [46]	USA	Mar 1, 2020	Mar 31, 2020	140	18 (12.9)	51.40	60.0 ± 6.9
Gayam V et al. [47]	USA	Mar 1, 2020	Apr 9, 2020	408	132 (32.4)	56.60	66.5 ± 5.8
Harmouch F et al. [48]	USA	Mar 1, 2020	Apr 15, 2020	560	81 (14.5)	57.10	63.5 ± 21.2
Hu H et al. [49]	China	Feb 7, 2020	Mar 7, 2020	105	19 (18.1)	50.90	60.8 ± 16.3
Huang J et al. [50]	China	Jan 25, 2020	Mar 24, 2020	299	16 (5.4)	53.50	53.4 ± 16.7
Hwang JM et al. [51]	South Korea	Feb 1, 2020	Mar 25, 2020	103	26 (25.2)	50.00	67.6 ± 15.3
Khalil K et al. [52]	UK	Mar 7, 2020	7 Apr, 2020	220	58 (26.4)	59.10	66.9 ± 17.0
Klang E et al. [53]	USA (≤50y)	Mar 1, 2020	May 17, 2020	572	60 (10.5)	69.40	40.7 ± 3.9
	USA (>50y)	Mar 1, 2020	May 17, 2020	2834	1076 (37.9)	55.20	71.1 ± 6.1
Krishnan S et al. [54]	USA	Mar 10, 2020	Apr 15, 2020	152	92 (60.5)	62.50	66.0 ± 13.0
Laguna-Goya R et al. [55]	Spain	Mar 10, 2020	Apr 12, 2020	501	36 (7.2)	63.30	52.0 ± 4.6
Li Q et al. [56]	China	Jan 20, 2020	Apr 4, 2020	1449	122 (8.4)	51.00	55.5 ± 6.9
Lieberman-Cribbin W et al. [57]	USA	Feb 29, 2020	Apr 24, 2020	6245	1128 (18.1)	NA	NA
Liu Q et al. [58]	China	Feb 1, 2020	Mar 13, 2020	336	34 (10.1)	50.30	62.5 ± 5.2
Long H et al. [59]	China	Jan 18, 2020	Mar 5, 2020	115	23 (20.0)	57.40	63.6 ± 13.9
Luo M et al. [60]	China	Jan 9, 2020	Mar 31, 2020	1018	201 (19.7)	51.20	60.0 ± 5.8
Luo X et al. [61]	China	Jan 30, 2020	Feb 20, 2020	289	84 (29.1)	50.30	55.8 ± 8.4
Luo Y et al. (a) [62]	China	Feb, 2020	Apr, 2020	739	51 (6.9)	49.40	60.1 ± 15.2
Luo Y et al. (b) [63]	China	Feb, 2020	Apr, 2020	1115	129 (11.6)	50.50	59.9 ± 15.3
Masetti C et al. [64]	Italy	Feb 28, 2020	Apr 10, 2020	229	33 (14.4)	64.60	60.7 ± 14.2
Mikami T et al. [65]	USA	Mar 12, 2020	Apr 17, 2020	2820	806 (28.6)	54.50	65.5 ± 9.1
Okoh AK et al. [66]	USA	Mar 10, 2020	Apr 10, 2020	251	97 (38.6)	51.00	61.8 ± 7.2
Pan F et al. [67]	China	Jan 27, 2020	Mar 19, 2020	124	89 (71.8)	68.50	66.9 ± 5.5
Rastad H et al. [68]	Iran	Feb 20, 2020	Mar 25, 2020	2957	301 (10.2)	53.70	54.8 ± 16.9
Richardson S et al. [69]	USA (18-65y)	Mar 1, 2020	Apr 4, 2020	1175	87 (7.4)	62.3	63.3 ± 6.6
	USA (>65y)	Mar 1, 2020	Apr 4, 2020	1425	466 (32.7)	57.1	
Rivera-Izquierdo M et al. [70]	Spain	Mar 16, 2020	Apr 10, 2020	238	61 (25.6)	55.00	64.7 ± 15.4
Ruan Q et al. [71]	China	Dec 31, 2019	Jan 31, 2020	150	68 (45.3)	68.00	56.8 ± 15.0
Salacup G et al. [72]	USA	Mar 1, 2020	Apr 24, 2020	242	52 (21.5)	51.00	66.5 ± 5.2
Shah P et al. [73]	USA	Mar 2, 2020	May 6, 2020	522	92 (17.6)	41.80	62.0 ± 6.3
Shang Y et al. [74]	China	Jan 1, 2020	Mar 27, 2020	113	49 (43.4)	64.60	65.6 ± 4.8
Shi S et al. [75]	China	Jan 1, 2020	Feb 23, 2020	671	62 (9.2)	48.00	62.0 ± 6.3
Soares RCM et al. [76]	Brazil	Feb 29, 2020	Jun 11, 2020	1152	456 (39.6)	57.10	NA
Sun H et al. [77]	China	Jan 29, 2020	Mar 5, 2020	244	121 (49.6)	54.50	69.7 ± 3.7
Wang K et al. [78]	China	Jan 7, 2020	Feb 11, 2020	340	33 (9.7)	48.20	48.3 ± 15.4
Xu B et al. [79]	China	Dec 26, 2019	Mar 1, 2020	145	28 (19.3)	52.40	60.9 ± 6.5
Yan X et al. [80]	China	Jan 11, 2020	Mar 13, 2020	1004	40 (4.0)	49.10	61.3 ± 6.0
Yang Q et al. [81]	China	Jan 1, 2020	Feb 29, 2020	226	50 (22.1)	50.00	53.9 ± 17.1
Yang X et al. [82]	China	Dec, 2019	Feb 25, 2020	1476	238 (16.1)	52.60	57.6 ± 6.8
Ye W et al. [83]	China	Jan 1, 2020	Mar 16, 2020	349	52 (14.9)	49.60	53.5 ± 13.8
Yu C et al. [84]	China	Jan 14, 2020	Feb 28, 2020	1464	212 (14.5)	50.30	62.5 ± 5.8
Zhang JJ et al. [85]	China	Dec 29, 2019	Feb 16, 2020	289	49 (17.0)	53.30	56.0 ± 19.1
Zhou F et al. [86]	China	Dec 29, 2019	Jan 31, 2020	191	54 (28.3)	62.00	56.3 ± 6.1

**NA**: not available.

^a^ When the mean or median were available for nonsurvivors and survivors, the weighted mean age for the total sample was calculated. When only the median and interquartile range for the total sample were available, the mean age was calculated according to the method proposed by Hozo SP, Djulbegovic B and Hozo I (BMC Med Res Methodol. 2005, 5:13).

Subjects with dyspnoea (p-OR = 2.5) and current smokers (p-OR = 1.6) were more likely to die than those without these conditions. All comorbidities were significantly associated with nonsurvival (p-ORs from 1.8 to 4.7). Headache (p-OR = 0.5), diarrhoea (p-OR = 0.6), vomiting (p-OR = 0.6), cough (p-OR = 0.7) and fever (p-OR = 0.8) were associated with a significantly lower risk of death. Moderate or high heterogeneity was detected in the analysis of almost all clinical symptoms and comorbidities (**Table 2**). All forest plots are available in the (**S3 Appendix**).

**Table 2:** Meta-analyses of sociodemographic and clinical potential predictors of in-hospital mortality.

Potential predictor	Studies, n	Total patients, n	Non-survivors, n (% exposed)	Survivors, n (% exposed)	Pooled odds ratio (95% CI)	p-value	I^2^
**Symptoms and signs** (yes/no)							
Dyspnoea	10	7577	1545 (63.5)	6032 (40.4)	2.54 (1.84, 3.50)*	<0.001	80.8
Fatigue	9	5884	954 (35.1)	4930 (22.1)	1.30 (0.59, 2.87)	0.52	94.4
Fever	17	10665	2069 (63.3)	8596 (63.5)	0.78 (0.64, 0.95)*	0.013	53.2
Myalgia	9	6608	1114 (19.2)	5494 (22.3)	0.77 (0.36, 1.64)	0.50	92.8
Cough	15	11911	2121 (62.6)	9790 (67.3)	0.74 (0.61, 0.91)*	0.003	63.7
Vomiting	6	5361	879 (7.7)	4482 (10.3)	0.60 (0.40, 0.89)*	0.011	32.0
Diarrhoea	12	9491	1846 (10.0)	7645 (15.8)	0.61 (0.45, 0.82)*	0.001	54.3
Headache	9	7384	1417 (8.6)	5967 (14.2)	0.52 (0.30, 0.90)*	0.020	74.9
**Chronic condition** (yes/no)							
Obesity	17	20289	6885 (14.2)	13404 (17.1)	1.09 (0.84, 1.41)	0.53	82.9
Smoking habit	23	18844	4436 (19.3)	14408 (16.2)	1.55 (1.24, 1.96)*	<0.001	73.1
Unspecified comorbidity	13	7644	1412 (77.7)	6232 (43.6)	4.70 (3.19, 6.91)*	<0.001	77.3
Stroke	5	1310	228 (14.9)	1082 (4.3)	4.15 (1.80, 9.59)*	0.001	45.5
Kidney disease	30	25413	7746 (14.8)	17667 (6.7)	3.20 (2.52, 4.06)*	<0.001	75.8
Cardiovascular disease	32	27052	7642 (30.8)	19410 (14.0)	2.98 (2.51, 3.53)*	<0.001	75.9
Hypertension	37	21388	4963 (61.4)	16425 (39.8)	2.61 (2.19, 3.17)*	<0.001	79.0
Malignancy	22	12687	2635 (15.8)	10052 (6.9)	2.36 (1.77, 3.15)*	<0.001	54.3
Diabetes	38	25498	5673 (33.8)	19825 (20.4)	2.12 (1.79, 2.52)*	<0.001	77.9
Pulmonary disease	31	24748	7848 (13.3)	16900 (7.5)	2.00 (1.39, 2.88)*	<0.001	88.0
Liver disease	13	13617	4432 (2.5)	9185 (2.7)	1.80 (1.35, 2.39)*	<0.001	6.2

* p-value <0.05.

Both survivors and nonsurvivors COVID-19 patients presented abnormal values for haemoglobin, D-dimer, albumin and all inflammatory factors. On the other hand, lymphocytes and neutrophil count, urea nitrogen and aspartate transaminase (AST) were out of the reference range only in nonsurvivors. Normal values were observed for both groups for leucocyte and platelet count, prothrombin time, activated partial thromboplastin time (APTT), total bilirubin, alanine transaminase (ALT) and creatinine (**Table 3**). The pooled mean showed worse values for all laboratory parameters in nonsurvivors, except for APTT, for which no significant difference was found between survival groups. Finally, the highest pooled effect sizes were observed for albumin (p-ES = -2.6), C-reactive protein (CRP) (p-ES = 2.5), urea nitrogen (p-ES = 2.4), interleukin-6 (p-ES = 2.3), neutrophil count (p-ES = 2.2), lactate dehydrogenase (LDH) (p-ES = 2.2), and lymphocyte count (p-ES = -2.0). The heterogeneity between studies for all laboratory parameters was high (I^2^ >90.0%) (**Table 3**).

**Table 3:** Meta-analyses of body mass index and laboratorial potential predictors of in-hospital mortality.

Potential predictor	Reference range	Studies, n	Non-survivors		Survivors		Effect estimates			
			Patients, n	Pooled Mean	Patients, n	Pooled Mean (95% CI)	Pooled Mean difference (95%CI)	Effect size (95% CI)	p-value	I^2^
				(95% C I)						
BMI (kg/m^2^)	18.5–24.9	6	1237	27.54	2887	27.88	-0.13	-0.01	0.925	89.1
				(26.91, 28.18)		(26.73, 29.03)	(-0.70, 0.44)	(-0.29, 0.27)		
**Routine blood tests**										
Lymphocyte count(x10^9^/L)	1.0–4.0	26	3121	0.69	12876	1.06	-0.36	-1.97	<0.001	99.3
				(0.57, 0.82)		(0.91, 1.21)	(-0.52, -0.20)	(-2.55, -1.38)*		
Leucocyte count (x10^9^/L)	3.6–11.0	26	2515	8.56	10959	5.92	2.75	1.79	<0.001	98.6
				(8.09, 9.03)		(5.06, 6.23)	(2.02, 3.49)	(1.32, 2.25)*		
Neutrophil count (x10^9^/L)	1.8–6.3	19	2195	7.18	9313	4.53	2.69	2.20	<0.001	99.0
				(6.77, 7.59)		(3.75, 5.30)	(1.89, 3.48)	(1.57, 2.83)*		
Platelet count (x10^9^/L)	140.0–400.0	18	1927	174.81	6917	212.04	-36.06	-1.37	<0.001	97.7
				(162.65, 186.96)		(200.81, 223.28)	(-49.34, -22.77)	(-1.78, -0.95)*		
Haemoglobin (g/L)	Men 140–170	16	1096	127.56	8798	130.84	-3.47	-0.41	0.018	97.2
	Women 120–150			(125.83, 129.28)		(127.91, 133.76)	(-5.84, -1.09)	(-0.75, -0.07)*		
**Coagulation**										
D-dimer (mg/L)	0–0.5	27	2460	4.17	8804	0.91	3.22	1.76	<0.001	97.6
				(3.65, 4.70)		(0.73, 1.09)	(2.84, 3.61)	(1.39, 2.13)*		
Prothrombin time (s)	10.0–14.0	11	626	12.66	2345	11.67	0.93	1.35	<0.001	97.2
				(7.92, 17.40)		(7.54, 15.80)	(0.45, 1.40)	(0.73, 1.97)*		
APTT (s)	30–40	10	671	34.17	2020	33.50	0.79	0.28	0.112	91.9
				(31.38, 36.97)		(30.58, 36.42)	(-0.13, 1.71)	(-0.07, 0.63)		
**Liver function/aggression**										
Albumin (g/L)	3.5–5.5	17	1145	29.50	7222	34.20	-4.70	-2.63	<0.001	99.0
				(26.24, 32.76)		(31.15, 37.26)	(-6.14, -3.26)	(-3.45, -1.80)*		
Total bilirubin (mmol/L)	3.0–22.0	10	618	10.72	1908	8.97	1.86	0.79	<0.001	94.5
				(6.29, 15.15)		(6.64, 11.29)	(0.93, 2.78)	(0.34, 1.24)*		
AST (U/L)	14.0–36.0	21	2098	49.53	9518	34.43	17.41	1.61	<0.001	97.5
				(45.36, 53.69)		(31.50, 37.36)	(13.99, 20.83)	(1.22, 2.00)*		
ALT (U/L)	9.0–52.0	25	2373	32.45	10542	30.16	2.18	0.44	<0.001	96.3
				(30.18, 34.72)		(27.97, 32.34)	(0.09, 4.28)	(0.39, 0.49)*		
**Kidney function**										
Urea nitrogen (mmol/L)	2.5–6.1	14	798	12.06	3737	5.80	6.64	2.43	<0.001	96.7
				(10.33, 13.79)		(5.31, 6.29)	(5.19, 8.09)	(1.90, 2.97)*		
Creatinine (μmmol/L)	46.0–92.0	21	1442	90.09	8173	68.33	21.72	1.28	<0.001	96.8
				(66.44, 113.74)		(48.74, 87.92)	(16.72, 26.71)	(0.97, 1.59)*		
**Inflammatory factors**										
CRP (mg/L)	0.5–10.0	30	3322	133.18	13338	64.39	68.31	2.49	<0.001	98.9
				(112.77, 153.59)		(54.96, 73.82)	(53.11, 83.50)	(2.01, 2.96)*		
Interleukin-6 (pg/mL)	0–7	12	1734	73.87	5735	29.88	43.64	2.31	<0.001	99.1
				(57.99, 89.76)		(25.08, 34.68)	(30.92, 56.35)	(1.47, 3.15)*		
LDH (U/L)	140.0–280.0	19	2230	506.89	9667	328.02	180.26	2.21	<0.001	98.5
				(336.59, 677.18)		(216.07, 439.97)	(131.02, 229.51)	(1.70, 2.72)*		
Procalcitonin (ng/mL)	0–0.15	18	2045	0.63	6367	0.16	0.52	1.58	<0.001	97.9
				(0.52, 0.74)		(0.13, 0.19)	(0.42, 0.62)	(1.14, 2.02)*		
Ferritin (ng/L)	Men 24–336	13	1663	1396.19	4404	797.98	603.94	1.56	<0.001	98.2
	Women 11–307			(1229.84, 1562.55)		(685.52, 910.44)	(383.27, 824.60)	(1.00, 2.12)*		
Cardiac troponin-I (ng/mL)	0–0.04	15	1161	0.05	3852	0.07	0.02	0.91	0.022	98.9
				(0.05, 0.06)		(0.04, 0.10)	(0.02, 0.02)	(0.13, 1.70)*		
ESR (mm/h)	0–15	6	358	57.15	1369	48.12	9.57	0.34	<0.001	91.5
				(32.28, 82.02)		(35.05, 61.19)	(1.75, 17.39)	(0.22, 0.47)*		

* p-value <0.05.

**ALT**: alanine transaminase; **APTT**: activated partial thromboplastin time, **AST**: aspartate transaminase; **BMI**: body mass index; **CRP**: C-reactive protein; **ESR**: erythrocyte sedimentation rate, **LDH**: lactate dehydrogenase.

Subgroup analysis are presented in **Table 4**. The following predictors were associated with worse prognosis in all age, sex and health status subgroups: dyspnoea, unspecified comorbidity, cardiovascular disease, hypertension, diabetes, kidney disease, pulmonary disease and cancer, lymphocytes, leukocytes, neutrophils and platelets count, D-dimer, prothrombin time, AST, urea nitrogen, creatinine, C-reactive protein, Interleukine-6, LDH and procalcitonin. The clinical symptoms fatigue, fever and myalgia, and the laboratory indicators APTT and erythrocyte sedimentation rate (ESR) were not associated with mortality in any of the subgroups. Obesity was only associated with increased mortality in studies with fewer chronic or critical patients (p-OR = 1.8). When body mass index (BMI) was examined as a continuous variable, risk of death decreased as BMI increased (p-ES = -0.2) in studies with a mean age of the patients of >60 years (the corresponding p-ES was not calculated for younger patients as only one study was available in this group) (**Table 4**).

**Table 4:** Subgroup meta-analyses^a^ of sociodemographic and clinical potential predictors of in-hospital mortality according to the predominant age, sex and health condition of the patients.

Potential predictor	Predominant age						Predominant sex						Predominant health condition^b^					
	n	>60 years		n	≤60 years		n	≥60% Men		n	<60% Men		n	Poor		n	Regular	
**Symptoms and signs**																		
Dyspnoea	4	1.59	0.0	5	4.29	72.8	0	-		10	2.54	80.8	2	1.72	0.0	8	2.90	80.9
		(1.34, 1.89)*			(2.51, 7.34)*						(1.84, 3.50)*			(1.41, 2.10)*			(1.94, 4.35)*	
Fatigue	6	1.42	93.9	3	1.08	90.5	3	1.48	0.0	7	1.25	96.5	5	1.03	38.0	4	1.75	97.5
		(0.57, 3.53)			(0.30, 3.87)			(0.96, 2.29)			(0.47, 3.29)			(0.71, 1.49)			(0.33, 9.12)	
Fever	9	0.76	42.1	7	0.90	31.5	4	0.64	0.0	14	0.80	63.0	8	0.70	56.9	9	0.82	54.4
		(0.57, 1.00)			(0.69, 1.19)			(0.39, 1.03)			(0.64, 1.00)			(0.49, 1.00)			(0.63, 1.07)	
Myalgia	5	0.50	95.8	4	1.09	25.7	1	-	-	8	0.87	93.5	4	0.49	96.9	5	0.98	41.8
		(0.13, 1.87)			(0.75, 1.60)						(0.40, 1.87)			(0.10, 2.44)			(0.65, 1.47)	
Cough	7	0.80	59.8	7	0.77	68.4	1	-	-	14	0.74	65.9	4	0.85	61.4	11	0.72	76.1
		(0.59, 1.10)			(0.55, 1.07)						(0.60, 0.91)*			(0.52, 1.41)			(0.57, 0.91)*	
Headache	4	0.26	34.1	4	0.99	57.8	0	-	-	9	0.52	74.9	2	0.19	0.0	7	0.73	58.8
		(0.14, 0.49)*			(0.42, 2.37)						(0.30, 0.90)*			(0.13, 0.28)*			(0.41, 1.33)	
**Chronic condition**																		
Obesity	11	0.90	79.7	5	1.62	78.5	6	1.59	91.0	11	1.03	73.7	10	0.77	78.9	7	1.76	79.3
		(0.67, 1.20)			(0.92, 2.83)			(0.60, 4.25)			(0.82, 1.29)			(0.56, 1.06)			(1.19, 2.61)*	
BMI	5	-0.23	52.6	1	-	-	2	-0.05	0.0	4	0.05	92.6	3	-0.08	0.0	3	0.15	94.9
		(-0.36, -0.10)*						(-0.24, 0.14)			(-0.36, 0.46)			(-0.24, 0.08)			(-0.39, 0.69)	
Smoking	13	1.21	30.0	9	2.78	76.3	5	2.62	89.7	18	1.37	50.2	12	1.14	24.4	11	2.34	80.9
		(1.04, 1.41)*			(1.43, 5.39)*			(0.81, 8.47)			(1.14, 1.64)*			(0.95, 1.37)			(1.52, 3.59)*	
Unspecified comorbidity	8	5.08	80.6	5	3.90	58.8	7	3.38	45.9	7	5.98	87.9	9	5.04	77.7	4	3.61	52.6
		(2.83, 9.13)*			(2.47, 6.17)*			(2.32, 4.91)*			(3.17, 11.30)*			(3.06, 8.30)*			(2.11, 6.17)*	
Kidney disease	21	3.06	76.0	8	3.80	67.2	7	3.17	43.7	23	3.21	79.8	18	3.07	77.1	12	3.39	75.5
		(2.30, 4.08)*			(2.33, 6.19)*			(1.76, 5.71)*			(2.46, 4.18)*			(2.18, 4.32)*			(2.34, 4.92)*	
Cardiovascular disease	21	3.02	78.6	10	3.02	69.7	9	2.45	38.6	23	3.20	80.9	16	2.90	83.0	16	2.89	62.1
		(2.34, 3.91)*			(2.27, 4.03)*			(1.79, 3.37)*			(2.61, 3.91)*			(2.12, 3.97)*			(2.37, 3.52)*	
Hypertension	27	2.31	78.2	10	3.73	68.1	12	2.51	38.2	25	2.65	84.2	23	2.46	81.6	14	2.85	74.8
		(1.91, 2.80)*			(2.60, 5.35)*			(2.00, 3.16)*			(2.11, 3.32)*			(1.93, 3.14)*			(1.93, 3.14)*	
Malignancy	15	2.10	55.2	7	3.69	21.1	7	2.41	0.0	15	2.47	65.4	14	2.09	58.1	8	3.27	16.5
		(1.56, 2.83)*			(1.94, 7.00)*			(1.75, 3.33)*			(1.64, 3.72)*			(1.52, 2.88)*			(1.93, 5.53)*	
Diabetes	26	1.85	72.5	11	3.17	81.9	10	1.85	0.0	28	2.21	83.1	23	1.96	75.1	15	2.44	82.2
		(1.55, 2.20)*			(1.95, 5.15)*			(1.49, 2.28)*			(1.80, 2.73)*			(1.58, 2.43)*			(1.79, 3.33)*	
Pulmonary disease	20	1.83	41.8	9	3.10	58.8	10	1.88	0.0	20	2.17	64.9	15	1.94	42.7	16	2.11	93.1
		(1.44, 2.32)*			(1.95, 4.93)*			(1.42, 2.50)*			(1.66, 2.84)*			(1.45, 2.59)*			(1.15, 3.88)*	
**Routine blood tests**																		
Lymphocyte count	16	-2.00	98.7	11	-1.86	99.5	9	-1.63	96.1	18	-2.13	99.5	12	-1.96	98.8	15	-1.97	99.5
		(-2.53, -1.46)*			(-3.13, -0.59)*			(-2.15, -1.12)*			(-2.95, -1.31)*			(-2.83, -1.10)*			(-2.77, -1.16)*	
Leucocyte count	11	1.57	98.6	15	1.94	98.7	18	2.11	94.2	9	1.19	98.8	14	1.74	97.6	12	1.84	99.0
		(0.79, 2.36)*			(1.32, 2.57)*			(1.51, 2.70)*			(0.72, 1.65)*			(1.16, 2.32)*			(1.11, 2.57)*	
Neutrophil count	12	2.08	98.9	8	2.37	99.0	5	1.88	96.3	15	2.30	99.2	9	2.09	97.4	11	2.29	99.3
		(1.37, 2.80)*			(1.12, 3.62)*			(1.10, 2.66)*			(1.52, 3.09)*			(1.38, 2.79)*			(1.35, 3.22)*	
Platelet count	11	-1.16	96.4	7	-1.69	98.1	8	-0.78	88.5	10	-1.84	98.6	10	-1.11	96.5	8	-1.68	98.5
		(-1.56, -0.75)*			(-2.58, -0.80)*			(-1.13, -0.43)*			(-2.48, -1.19)*			(-1.66, -0.57)*			(-2.39, -0.97)*	
Haemoglobin	10	-0.29	94.3	6	-0.60	98.0	6	-0.44	96.2	10	-0.39	97.8	9	-0.30	95.9	7	-0.55	97.3
		(-0.82, 0.24)			(-1.02, -0.19)*			(-1.14, 0.27)			(-0.80, 0.03)			(-0.79, 0.19)			(-0.98, -0.12)*	
**Coagulation**																		
D-dimer	18	1.78	97.8	10	1.72	96.8	10	1.52	91.4	18	1.90	98.2	14	2.04	96.0	14	1.48	98.4
		(1.32, 2.24)*			(1.09, 2.35)*			(1.15, 1.89)*			(1.38, 2.42)*			(1.57, 2.51)*			(0.90, 2.06)*	
Prothrombin time	8	1.37	97.4	3	1.30	97.5	4	1.09	97.7	7	1.50	97.0	7	1.30	97.7	4	1.44	96.4
		(0.56, 2.18)*			(0.18, 2.43)*			(0.02, 2.16)*			(0.70, 2.29) *			(0.40, 2.19)*			(0.55, 2.33)*	
APTT	5	0.44	95.7	5	0.11	69.5	3	0.22	95.4	7	0.30	91.1	5	0.44	95.7	5	0.11	69.5
		(-0.31, 1.18)			(-0.13, 0.35)			(-0.66, 1.11)			(-0.09, 0.70)			(-0.31, 1.18)			(-0.13, 0.35)	
**Liver function/aggression**																		
Albumin	11	-3.05	98.9	6	-1.85	99.2	7	-2.16	98.1	10	-2.96	99.1	8	-2.73	98.8	9	-2.53	99.0
		(-4.11, -1.99)*			(-3.40, -0.30)*			(-3.14, -1.18)*			(-4.12, 1.79)			(-3.93, -1.53)*			(-3.67, -1.40)*	
Total bilirubin	6	0.66	80.8	4	0.98		6	0.85	96.2	4	0.69	88.4	5	0.59	79.4	5	0.98	97.0
		(0.35, 0.97)*			(-0.18, 2.14)			(0.14, 1.57)*			(0.20, 1.18)*			(0.26, 0.93)*			(0.11, 1.84)*	
AST	14	1.79	97.8	7	1.25	97.2	9	0.96	92.1	12	2.09	97.1	11	1.64	97.7	10	1.59	96.4
		(1.28, 2.29)*			(0.50, 2.00)*			(0.55, 1.38)*			(1.65, 2.53)*			(0.99, 2.30)*			(1.15, 2.02)*	
ALT	15	0.42	93.1	10	-0.01	97.9	10	-0.03	97.8	15	0.45		12	0.40	92.3	13	0.12	97.6
		(0.16, 0.68)*			(-0.66, 0.65)			(-0.73, 0.67)			(0.22, 0.67)*			(0.07, 0.73)*			(-0.30, 0.54)	
**Kidney function**																		
Urea nitrogen	9	2.66	97.4	5	2.03	94.8	5	2.31	93.5	9	2.51	97.6	7	2.63	97.8	7	2.25	94.7
		(1.92, 3.40)*			(1.25, 2.82)*			(1.62, 3.00)*			(1.73, 3.28)*			(1.71, 3.54)*			(1.63, 2.88)*	
Creatinine	14	1.30	94.4	8	2.42	98.1	8	1.21	93.3	14	1.86	97.5	12	1.76	97.3	10	1.46	94.8
		(0.95, 1.66)*			(1.56, 3.29)*			(0.75, 1.66)*			(1.34, 2.39)*			(1.19, 2.34)*			(1.02, 1.89)*	
**Inflammatory factors**																		
CRP	20	2.69	99.0	11	2.11	98.6	11	2.25	98.9	19	2.67	98.9	16	2.26	98.8	15	2.73	98.7
		(2.07, 3.32)*			(1.30, 2.93)*			(1.20, 3.29)*			(2.10, 3.24)*			(1.56, 2.96)*			(2.11, 3.35)*	
Interleukin-6	6	2.23	99.4	6	2.39	98.8	5	2.10	92.2	7	2.45	99.4	5	1.35	96.1	7	3.01	98.9
		(0.92, 3.54)*			(1.19, 3.59)*			(1.59, 2.61)*			(1.23, 3.67)*			(0.69, 2.00)*			(2.06, 3.97)*	
LDH	13	2.43	98.7	7	1.81	98.1	9	2.47	98.3	11	2.00	98.3	9	2.29	97.4	11	2.15	98.9
		(1.74, 3.12)*			(0.93, 2.69)*			(1.54, 3.40)*			(1.34, 2.66)*			(1.51, 3.06)*			(1.45, 2.85)*	
Procalcitonin	11	1.64	97.0	7	1.50	98.2	6	1.63	95.8	12	1.55	98.4	10	2.22	96.8	8	0.78	98.7
		(1.20, 2.07)*			(0.53, 2.46)*			(0.95, 2.31)*			(0.98, 2.13)*			(1.66, 2.79)*			(0.04, 1.52)*	
Ferritin	9	1.74	98.0	4	1.11	98.8	7	1.48	97.0	6	1.66	98.9	6	0.44	96.7	7	2.54	98.3
		(1.16, 2.32)*			(-0.80, 3.02)			(0.75, 2.21)*			(0.69, 2.62)*			(-0.23, 1.11)			(1.69, 3.40)*	
Cardiac troponin I	10	1.01	99.2	5	0.72	93.7	7	1.71	97.6	8	0.22	99.1	6	1.52	98.9	9	0.51	99.0
		(-0.11, 2.13)			(0.08, 1.36)*			(0.82, 2.59)*			(-0.92, 1.37)			(0.32, 2.73)*			(-0.61, 1.62)	
ESR	3	0.14	90.6	3	0.80	94.1	1	-	-	5	0.17	82.6	2	0.12	95.0	4	0.62	92.3
		(-0.36, 0.65)			(-0.32, 1.91)						(-0.17, 0.51)			(-0.80, 1.05)			(-0.09, 1.33)	

**ALT**: alanine transaminase; **APTT**: activated partial thromboplastin time, **AST**: aspartate transaminase; **BMI**: body mass index; **CRP**: C-reactive protein; **ESR**: erythrocyte sedimentation rate, **LDH**: lactate dehydrogenase.

* p-value <0.05, n: number of studies.

^a^ Values indicate the pooled risk estimators for each predictor (odds ratio for categorical and effect size for BMI and biochemical variables).

^b^ The “poor” group comprises studies in which all patients were critical or severe or the prevalence of any of the comorbidities was >50%.

The following predictors were more markedly associated with worse prognosis in studies with younger patients (mean age ≤60 years), a lower proportion of men (<60%) and better health (<50% with chronic/critical/severe condition): smoking, dyspnoea, hypertension, malignancy, diabetes, kidney and pulmonary disease, increased neutrophil and decreased platelet count, and increased interleukin-6. Decreased haemoglobin only predicted higher mortality in younger patients (p-ES = -0.6) and in those with better health (p-ES = -0.6). Increased creatinine levels showed worse prognosis value in younger patients (p-ES = 2.4) than in older patients (p-ES = 1.3), while the effect size for abnormal nitrogen urea was moderately higher in older patients (p-ES = 2.7) than in younger patients (p-ES = 2.0) (**Table 4**).

All indicators of liver function or aggression were more strongly associated with mortality in studies with older patients, as albumin (p-ES = -3.1 for older patients and -1.9 for younger patients) and aspartate transaminase (p-ES = 1.8 for older patients and 1.3 for younger patients). In the case of total bilirubin and alanine transaminase, small-sized effects were found only in studies with older patients (p-ES = 0.7 and 0.4, respectively) (**Table 4**).

Abnormal values of C-reactive protein, LDH, procalcitonin and ferritin were the inflammatory indicators with greater effect size in studies with older patients (p-ES = 2.7, 2.4, 1.6 and 1.7, respectively) than in those with younger patients (p-ES = 2.1, 1.8, 1.5 and 1.1, respectively). On the other hand, abnormal values of cardiac troponin I predicted worse prognosis only in studies with younger patients (p-ES = 0.7), a higher proportion of men (p-ES = 1.7) and worse health conditions (p-ES = 1.5) (**Table 4**).

Meta-regression showed that the mean age of patients is an important source of heterogeneity when examining the potential predictive effect of dyspnoea, BMI, smoking, hypertension, malignancy and pulmonary disease (**Table 5**).

**Table 5:** Meta-regression models adjusted for characteristics of the included studies according to the age, sex and health condition of the patients.

Potential predictor	Original I^2^	Age-adjusted model^a^	Sex-adjusted model^b^	Health-adjusted model^c^
		I^2^ residual	I^2^ residual	I^2^ residual
Dyspnoea	80.8	68.0*	83.0	78.4
Fatigue	94.4	94.8	94.4	77.7
Fever	53.2	33.5	41.7	55.9
Myalgia	92.8	93.6	91.4	93.3
Cough	63.7	64.9	65.7	50.4
Headache	74.9	73.3	73.9	0.0**
Obesity	82.9	79.9	84.0	80.3
BMI	89.1	75.0**	90.8	89.5
Smoking habit	73.1	62.0*	74.9	72.7
Unspecified comorbidity	77.3	76.9	74.6	67.8
Kidney disease	75.8	69.6	76.5	76.5
Cardiovascular disease	75.9	74.6	72.8	76.5
Hypertension	79.0	69.0**	77.9	79.2
Malignancy	54.3	8.7*	56.2	52.7
Diabetes	77.9	71.8	76.6	78.4
Pulmonary disease	88.0	40.9*	54.7	86.3
Lymphocyte count	99.3	99.2	99.3	99.4
Leucocyte count	98.6	98.6	98.4	98.6
Neutrophil count	99.0	98.9	98.9	98.9
Platelet count	97.7	96.9	96.3	98.2
Haemoglobin	97.2	97.4	97.4	96.2
D-dimer	97.6	97.5	97.6	97.8
Prothrombin time	97.2	97.4	96.9	97.7
APTT	91.9	91.5	92.8	94.6
Albumin	99.0	98.9	98.7	98.9
Total bilirubin	94.5	95.1	95.1	85.2
AST	97.5	97.6	95.9	97.6
ALT	96.3	96.4	95.8	96.4
Urea nitrogen	96.7	96.9	96.8	97.2
Creatinine	96.8	96.9	96.8	96.6
CRP	98.9	98.9	98.8	98.9
Interleukin-6	99.1	99.2	98.9	98.6
LDH	98.5	98.6	98.4	98.4
Procalcitonin	97.9	97.6	98.0	97.9
Ferritin	98.2	98.3	98.3	96.9
Cardiac troponin-I	98.9	98.9	98.7	98.4
ESR	91.5	93.2	54.6**	90.6

* p-value <0.05

** p-value <0.01.

**ALT**: alanine transaminase; **APTT**: activated partial thromboplastin time, **AST**: aspartate transaminase; **BMI**: body mass index; **CRP**: C-reactive protein; **ESR**: erythrocyte sedimentation rate, **LDH**: lactate dehydrogenase.

^a^ Meta-regression model adjusted for the estimated mean age (y) of each study. When the mean or median were available for nonsurvivors and survivors, the weighted mean age for the total sample was calculated. When only the median and interquartile range for the total sample were available, the mean age was calculated according to the method proposed by Hozo SP, Djulbegovic B and Hozo I (BMC Med Res Methodol. 2005, 5:13).

^b^ Meta-regression model adjusted for the proportion of male sex in each study.

^c^ Meta-regression model adjusted for the highest prevalence of chronic conditions or critical/severe status of patients in each study.

The pooled-ES estimates were not substantially modified when the data of individual studies were removed from the analysis one at a time. No relevant differences were observed in sensitivity analyses when studies with moderate risk of bias in more than one domain of the QUIPS tool were excluded, as presented in **S2** and **S3 Tables**.

There was evidence of publication bias in both funnel plot asymmetry and Egger’s test for cough, unspecified comorbidity, cardiovascular disease, hypertension, malignancy, diabetes, haemoglobin, prothrombin time, urea nitrogen and C-reactive protein. All funnel plots and Egger’s test p-values are available in the (**S3 Appendix**).

## Discussion

This meta-analysis included 60 studies with primary data from more than 50,000 patients with confirmed COVID-19, of whom a quarter died. Higher in-hospital mortality risk was found for patients with dyspnoea; a smoking habit; pulmonary, cardiovascular, cerebrovascular, kidney and liver diseases; hypertension; diabetes; and malignancy. Among laboratory parameters, the highest mortality risk was observed for routine blood tests (lymphocyte, leucocyte, neutrophil and platelet counts and haemoglobin), coagulation indicators (D-dimer and prothrombin time), liver function and aggression markers (albumin, total bilirubin, aspartate and alanine transaminase), kidney function markers (urea nitrogen and creatinine), and inflammatory factors (CRP, IL-6, LDH, procalcitonin, ferritin and cardiac troponin). In subgroup analyses and meta-regression, age was the main source of heterogeneity, followed by sex and health condition.

Age played an important role in subgroup analyses. For instance, the increased mortality risks were higher in studies with younger patients than those in studies with older adult patients regarding dyspnoea (p-OR = 4.3 vs. 1.6) and smoking (p-OR = 2.8 vs. 1.2). Additionally, the risks associated with comorbidities were also higher among younger populations, in agreement with the findings of a previous meta-analysis on hypertension [11]. Sex differences may be considered, since smoking increased the risk of death only in studies with lower proportion of male patients. It has been suggested that this could be due to the higher prevalence of chronic conditions and smoking and poorer immunological status in men [87]. Moreover, smoking is more frequent in men than in women, and smokers usually have chronic respiratory conditions, and dyspnoea is a frequent symptom [88]. Although this cluster effect may explain part of the increased risk in smokers, it has been suggested that nicotine can directly affect the putative virus receptor (ECA2) and lead to harmful signalling in the lung epithelial cells, which may contribute to worse prognosis of respiratory viral infections [89]. The interpretation of these findings may indicate that dyspnoea and smoking are more markedly associated with worse prognosis in populations with less exposure to the main risk factors for mortality in COVID-19, that is, populations with advanced age, a high proportion of men and a high prevalence of comorbidities.

Among the symptoms and clinical signs studied, only dyspnoea was associated with higher mortality risk in this meta-analysis. Surprisingly, this risk was reduced in the presence of fever, cough, vomiting, diarrhoea and headache, which are the most prevalent symptoms in COVID-19 [3, 4, 90]. Other meta-analyses found only borderline [8] or no significant association between these symptoms and mortality [10], although all included less studies than the present meta-analysis. We cannot explain these unexpected relationships, although some of them have been reported elsewhere [91]. However, it could be hypothesized that the reporting of these symptoms is affected by information bias and may include single variable symptoms that are mild and occasional (characteristic of mild cases of the disease) or strong and persistent (justifying a worse prognosis given the advanced stage of the disease). Additionally, these associations were not consistent in the subgroup analyses, reinforcing that these symptoms, unlike dyspnoea, may not differentiate patients with worse prognosis. Pulmonary impairment results in low ventilation and oxygenation, which is expressed clinically by dyspnoea [92]. Although dyspnoea is frequent in COVID-19 patients [93–95], its detection on hospital admission highlights the need for specific attention compared with patients without this respiratory symptom, particularly if they are younger adults.

In the present study, a higher mortality risk was observed for all studied comorbidities. The overall risk estimators we found are similar to those of other meta-analyses for smoking [96, 97]; dyspnoea [8]; pulmonary [10, 96, 98], cardiovascular [8, 10, 98, 99], cerebrovascular [10, 99], kidney [10] and liver diseases [17]; hypertension [10, 98]; diabetes [10, 98, 100]; and malignancy [101]. In addition to confirming that chronic conditions lead to worse prognosis, the present study suggests that the association with worse prognosis is of greater magnitude for patients with lower mean age, lower percentage of men and lower prevalence of chronic or critical health condition, although they are also important predictors of mortality in the other subgroups. Similarly, obesity was associated with increased mortality only in studies with fewer chronic or critical patients. Two previous meta-analyses found significant associations between obesity and increased risk of adverse or severe outcomes, although mortality was not specifically addressed [102, 103]. Thus far, current evidence suggests that excess weight and obesity are relevant to adverse COVID-19 outcomes [104–107]. However, our results suggest that BMI is probably a more prominent prognostic factor in patients with fewer comorbidities.

Abnormal levels of laboratory parameters common in infectious conditions were especially associated with mortality when combining the results of the studies included in this review. Haemoglobin, D-dimer, albumin and all inflammatory factors were impaired in all patients, while lymphocyte and neutrophil counts, AST and urea nitrogen were impaired only for non-survivors. The highest risk was observed for the inflammatory factors, albumin and kidney function indicators, although significant effect sizes were observed for all parameters included in this meta-analysis, except for APTT, results consistent with previous meta-analyses [108–110]. The fact that APTT is not a good marker of COVID-related poor coagulation suggests that D-dimer and prothrombin time may be more sensitive indicators for regulating the anticoagulant doses in these patients [111].

In subgroup analyses, the most important mortality predictors in studies with older patients were liver function-related indicators (albumin, total bilirubin, AST and ALT), urea nitrogen and inflammatory factors including C-reactive protein, LDH and ferritin. Likewise, other authors observed that, compared to younger patients, older COVID-19 patients had higher levels of AST, ALT, C-reactive protein and LDH [90], which illustrates the potential for more serious organ damage caused by SARS-CoV-2 infection in the elderly. According to our results, high values in these specific biochemical markers could be used for early identification of elderly patients with worse prognosis. On the other hand, factors noted in younger patients included decreased platelet count, decreased haemoglobin, increased creatinine, increased interleukin-6 and increased cardiac troponin I. Although reduced haemoglobin was related to worse prognosis in the main analysis, confirming previous results [110], this association was detected only in low-risk populations in the subgroup analysis (younger and healthier patients). The significant effect (p-ES = -0.6) found for these associations indicates that even small reductions in haemoglobin may identify those with the lowest chance of survival among these patients. Future studies are needed to examine whether differences in the magnitude of effect sizes point to age-specific indicators for worse prognosis of SARS-CoV-2 infection and whether these variations are due to other variables not available in the included studies to explain the high and persistent heterogeneity between them, such as social and cultural factors related to gender rather than biological sex factors [13].

Some methodological aspects must be considered when interpreting the results presented. First, all included studies had a retrospective design, which is related to some concerns regarding the risk of bias in the selection of participants and in the identification of the outcome [112]. In addition, they were based on medical records fulfilled in a period during which the hospitals were likely to be overwhelmed due to the increased demand caused by SARS-CoV-2 infections, which could result in some underreporting or even inaccuracy of important data [113, 114]. Second, studies included in this review included only in-hospital patients, among whom the intrinsic mortality risk is higher. Third, moderate and high heterogeneity was observed in the meta-analysis of almost all risk factors; however, we could not explore most of the results in subgroup analyses, as there were limited available data in the included studies. Finally, publication bias was observed in one of four of the studied predictors. This is a limitation shared with previous meta-analyses [10, 115] and it indicates that some specific results should be confirmed when more studies are available.

In addition to the clinical implications resulting from the precise identification of predictors of COVID-19-related mortality with suggestions for possible specificities according to the profile of the patients, this systematic review also has implications for future studies. For example, as the evidence indicates that COVID-19 is potentially more dangerous for elderly populations and men, we emphasize that the following studies should present primary data separated according to age and sex groups, allowing the assessment of specific risks according to these characteristics [13]. Last, other potential risk factors not included in this review may be relevant for patients with different demographic and epidemiological profiles. For example, some emerging evidence indicates that the prevalence of rigors, wheeze and hyposmia [3, 116] is high in COVID-19 patients.

In conclusion, this study provides robust evidence on the relative importance of age, sex and health conditions of the patients to the prognostic value of clinical and biochemical parameters observed in COVID-19 patients around the world. Therefore, epidemiological data stratified by age, sex and baseline chronic conditions are required to allow for more accurate decisions considering predictors of COVID-mortality.

## Supporting information

S1 Checklist MOOSE (meta-analyses of observational studies in Epidemiology) checklist. (PDF)

S1 Appendix Full search strategy. (PDF)

S2 Appendix Chinese studies with potential overlapping of patients’ data. (PDF)

S3 Appendix Forest plots, funnel plots and Egger’s test p-values. (PDF)

S1 Table Description of sub-groups and corresponding criteria. (PDF)

S2 Table Risk of bias assessment of the included studies. (PDF)

S3 Table Main meta-analyses results obtained in sensitivity analyses excluding studies with moderate risk of bias in more than one domain. (PDF)
