# Supplementary material for: Predictors of in-hospital COVID-19 mortality: A comprehensive systematic review and meta-analysis exploring differences by age, sex and health conditions
Source: PLoS One. 2020 Nov 3;15(11):e0241742. doi: 10.1371/journal.pone.0241742 (PMC7608886; doi:10.1371/journal.pone.0241742)
Supplement: S1 Appendix — (PDF) [file pone.0241742.s002.pdf]

Supporting Information

Predictors of in-hospital COVID-19 mortality: a comprehensive systematic review and meta-analysis exploring differences by age, sex and health conditions

S1 Appendix: Full search strategy

Database	Syntaxis
PubMed	(coronavirus OR Covid-19 OR "Covid 19" OR Covid19 OR 2019-nCoV OR SARS-CoV-2 OR SARS-CoV2) AND (mortality OR death OR died OR surviv* OR decease* OR fatal*)
Scopus	ALL(mortality OR death OR died OR surviv* OR decease* OR fatal*) AND ALL(coronavirus OR Covid-19 OR "Covid 19" OR Covid19 OR 2019-nCoV OR SARS-CoV-2 OR SARS-CoV2)
Web of Science	TS=(mortality OR death OR died OR surviv* OR decease* OR fatal*) AND TS=(coronavirus OR Covid-19 OR "Covid 19" OR Covid19 OR 2019-nCoV OR SARS-CoV-2 OR SARS-CoV2)
