# Supplementary material for: Predictors of in-hospital COVID-19 mortality: A comprehensive systematic review and meta-analysis exploring differences by age, sex and health conditions
Source: PLoS One. 2020 Nov 3;15(11):e0241742. doi: 10.1371/journal.pone.0241742 (PMC7608886; doi:10.1371/journal.pone.0241742)
Supplement: S2 Appendix — (PDF) [file pone.0241742.s003.pdf]

Supporting Information

Predictors of in-hospital COVID-19 mortality: a comprehensive systematic review and meta-analysis exploring differences by age, sex and health conditions

S2 Appendix: Chinese studies with potential overlapping of patients' data.

When more than one study provided data for one specific predictor from patients from the same hospital and time interval, we only considered the study with later end date. The lists below indicate the order of preference according to this criterion.

Tongji Hospital

1	Luo Y et al. (b)	Feb, 2020 – Apr, 2020
2	Luo Y et al. (a)	Feb, 2020 – Apr, 2020
3	Luo M et al.	Jan 9, 2020 – Mar 19, 2020
4	Cheng A et al.	Feb 8, 2020 – Mar 11, 2020
5	Sun H et al.	Jan 29, 2020 – Mar 5, 2020
6	Yu C et al.	Jan 14, 2020 – Feb 28, 2020
7	Chen T et al.	Jan 13, 2020 – Feb 28, 2020
8	Deng Y et al.	Jan 1, 2020 – Feb 21, 2020
9	Ruan Q et al.	Dec 31, 2019 – Jan 31, 2020

Central Hospital of Wuhan

1	Li Q et al.	Jan 20, 2020 – Apr 4, 2020
2	Yang Q et al.	Jan 1, 2020 – Feb 29, 2020
3	Deng Y et al.	Jan 1, 2020 – Feb 21, 2020
4	Chen F et al.	Jan 1, 2020 – Feb 15, 2020

Wuhan Pulmonary Hospital

1	Luo M et al.	Jan 9, 2020 – Mar 19, 2020
2	Ye W et al.	Jan 1, 2020 – Mar 16, 2020
3	Du RH et al.	Dec 25, 2019 – Feb 7, 2020
4	Zhou F et al.	Dec 31, 2019 – Jan 31, 2020

Jinyintan Hospital

1	Li Q et al.	Jan 20, 2020 – Apr 4, 2020
2	Yang X et al.	Late Dec, 2019 – Feb 25, 2020
3	Zhou F et al.	Dec 31, 2019 – Jan 31, 2020
4	Ruan Q et al.	Dec 31, 2019 – Jan 31, 2020

Zhongnan Hospital

1	Shang Y et al.	Jan 1, 2020 - Mar 27, 2020

2	Zhang JJ et al.	Dec 29, 2019 - Feb 16, 2020
3	Cao J et al.	Jan 3, 2020 - Feb 1, 2020

Union Hospital

1	Li Q et al.	Jan 20, 2020 – Apr 4, 2020
2	Pan F et al.	Jan 27, 2020 – Mar 19, 2020
3	Liu Q et al.	Feb 1, 2020 – Mar 13, 2020

Renmin Hospital

1	Shi S et al.	Jan 1, 2020 – Feb 23, 2020
2	Luo X et al.	Jan 30, 2020 – Feb 20, 2020

Wuhan Third Hospital

1	Li Q et al.	Jan 20, 2020 – Apr 4, 2020
2	Gao S et al.	Jan 23, 2020 – Feb 29, 2020

First People's Hospital of Jiangxia District

1	Wang K et al.	Jan 7, 2020 – Feb 11, 2020

Hubei Provincial Hospital of Traditional Chinese and Western Medicine,

1	Xu B et al.	Dec 26, 2019 – Mar 1, 2020

Third People's Hospital of Yichang

1	Huang J et al.	Jan 25, 2020 – Mar 24, 2020

Tianyou Hospital

1	Long H et al.	Jan 18, 2020 – Mar 5, 2020

Tongren Hospital of Wuhan University

1	Yan X et al.	Jan 11, 2020 – Mar 3, 2020

Obs.: Hu H et al. did not inform the name of the hospital which data came from.
