# Supplementary material for: Predictors of in-hospital COVID-19 mortality: A comprehensive systematic review and meta-analysis exploring differences by age, sex and health conditions
Source: PLoS One. 2020 Nov 3;15(11):e0241742. doi: 10.1371/journal.pone.0241742 (PMC7608886; doi:10.1371/journal.pone.0241742)
Supplement: S3 Appendix — (PDF) [file pone.0241742.s004.pdf]

Supporting Information

Predictors of in-hospital COVID-19 mortality: a comprehensive systematic review and meta-analysis exploring differences by age, sex and health conditions.

S3 Appendix: Forest plots, funnel plots and Egger's test p-values.

(Please, find the corresponding information on the following pages)

Dyspnoea

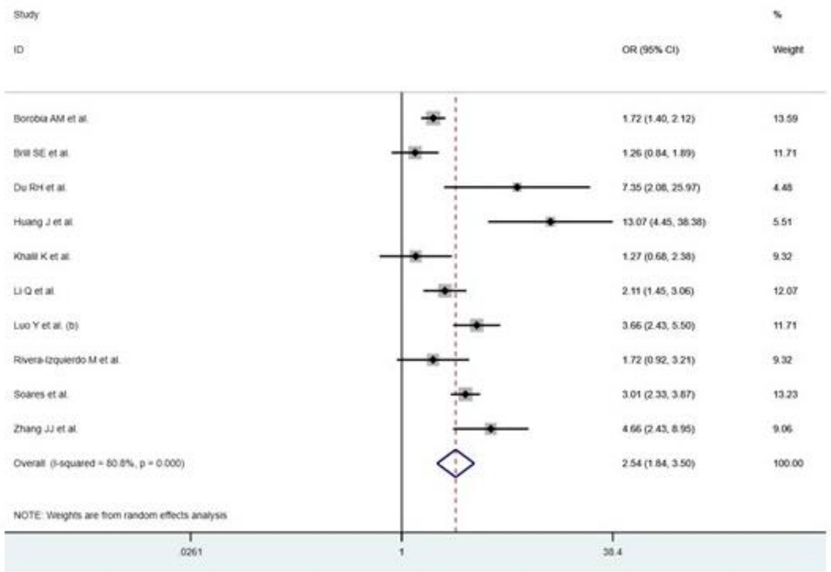

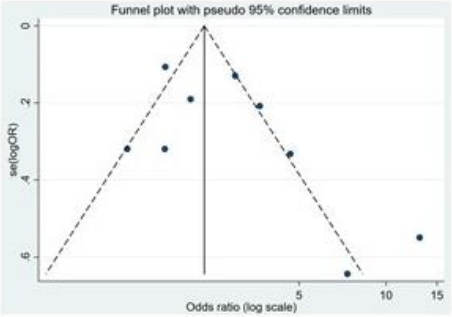

Egger's test: p-value=0.17

Vomiting

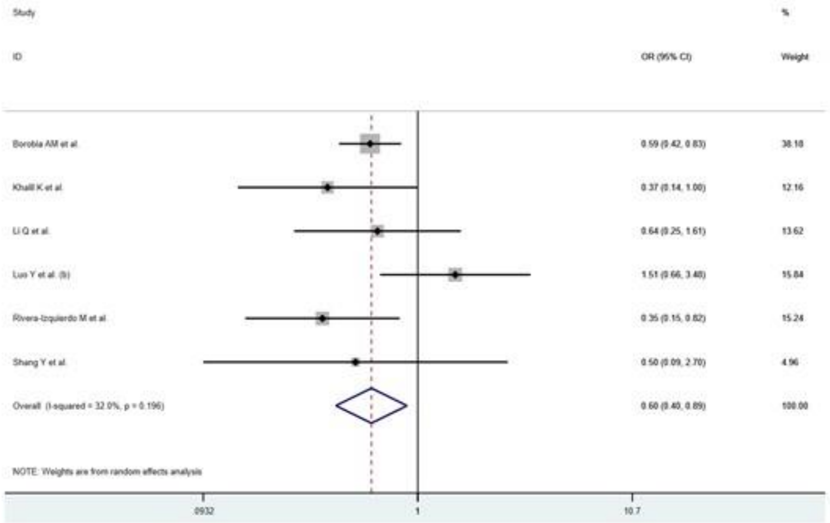

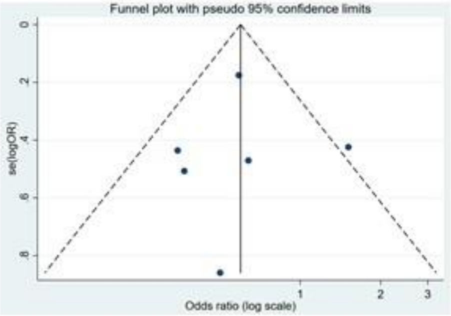

Egger's test: p-value=0.93

Fatigue

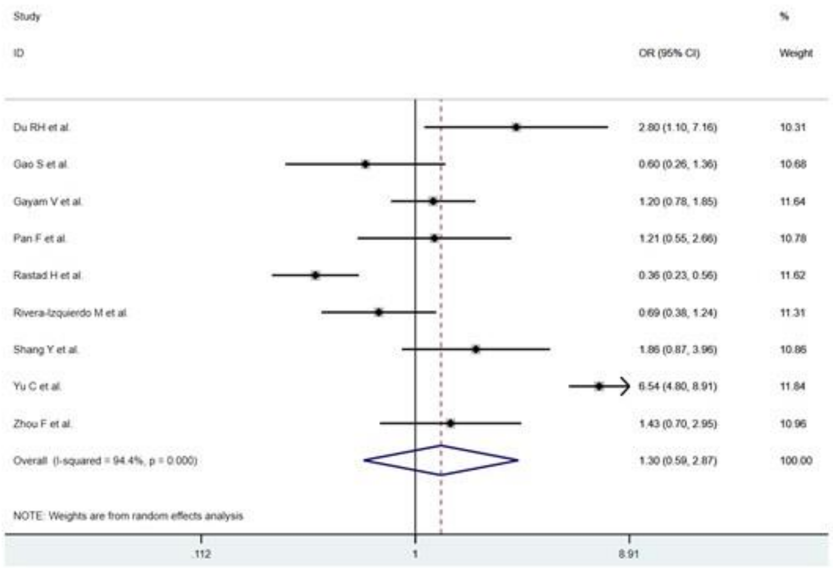

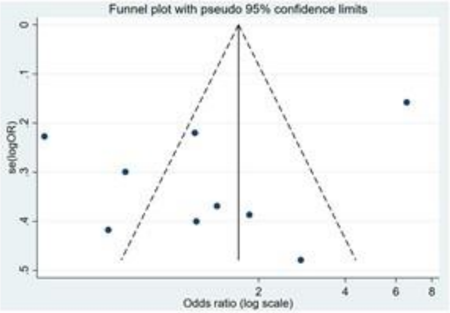

Egger’s test: p-value=0.25

Fever

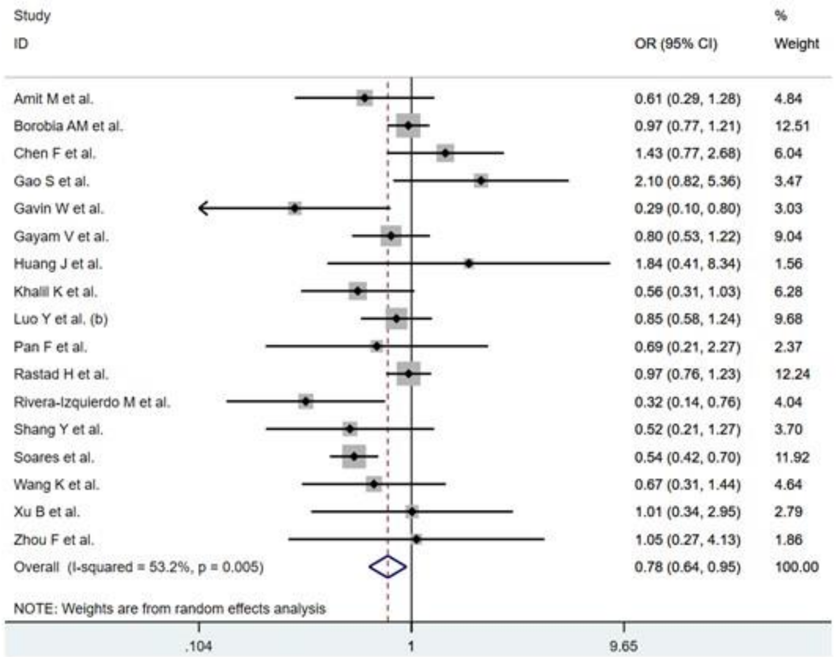

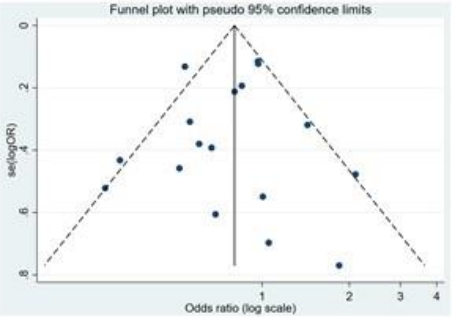

Egger's test: p-value=0.68

Myalgia

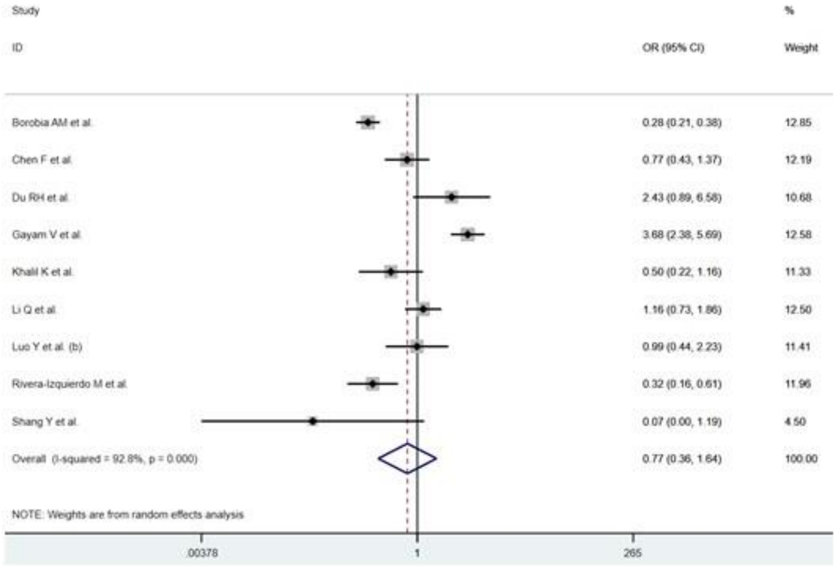

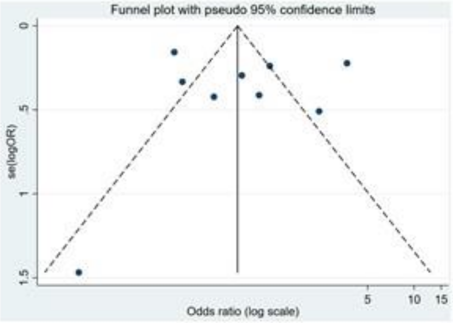

Egger's test: p-value=0.74

Cough

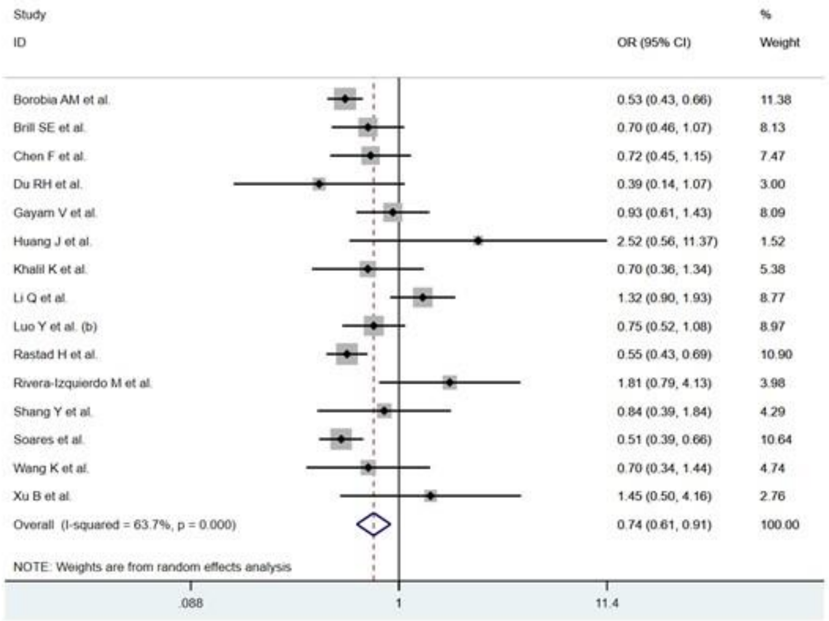

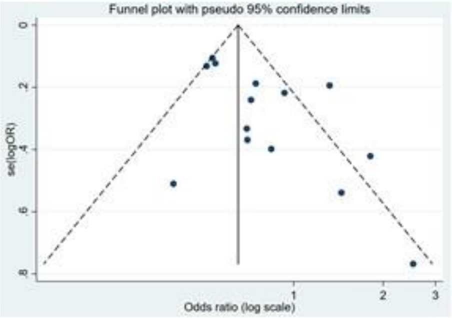

Egger's test: p-value=0.022

Diarrhoea

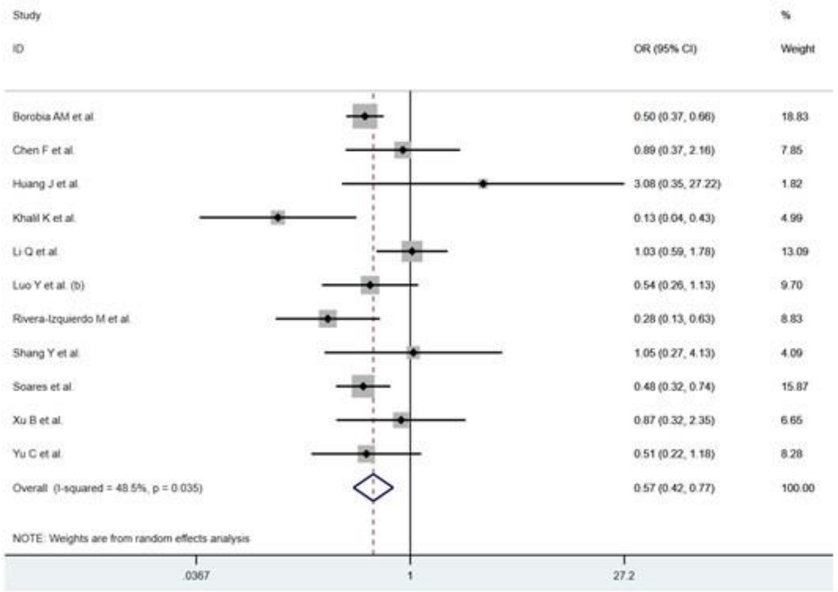

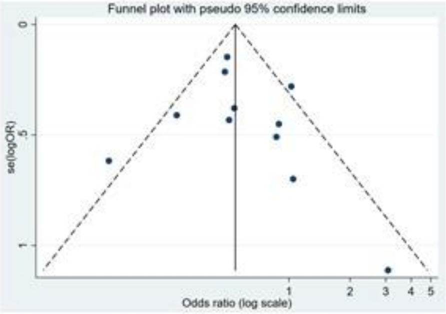

Egger’s test: p-value=0.56

Headache

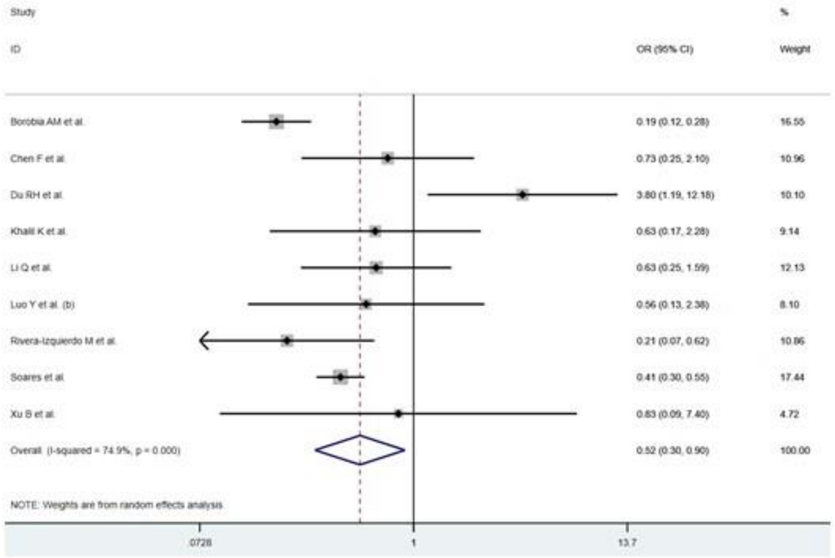

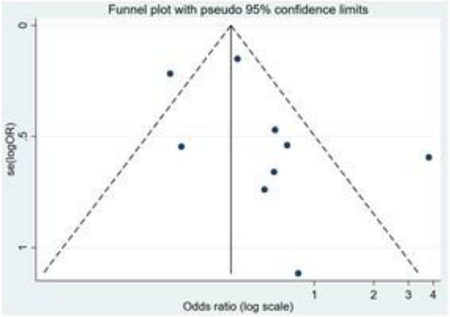

Egger's test: p-value=0.22

Obesity

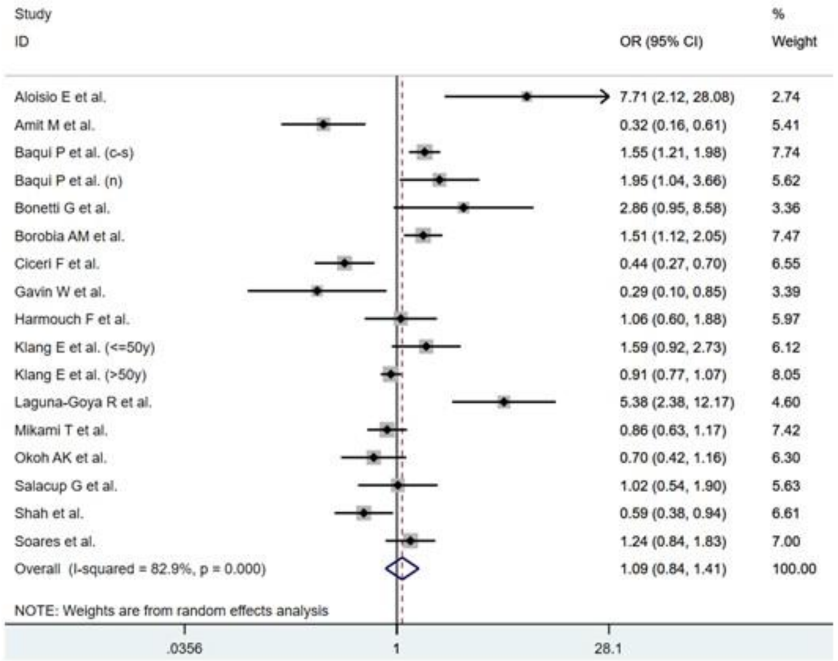

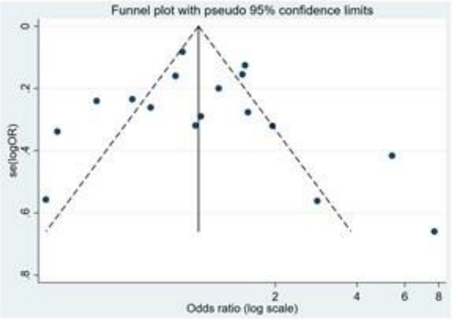

Egger's test: p-value=0.66

BMI

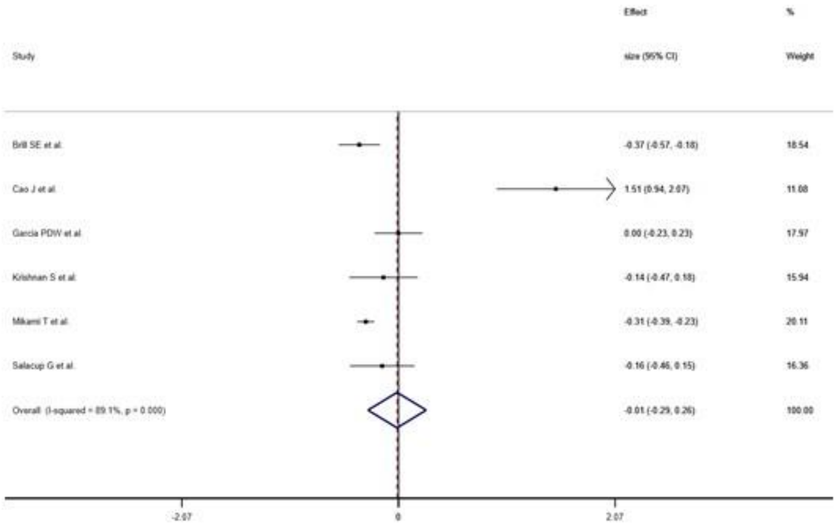

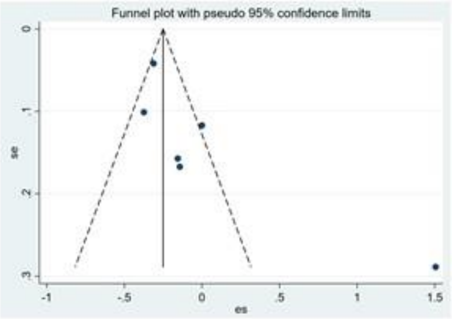

Egger's test: p-value=0.103

Smoking habit (yes/no)

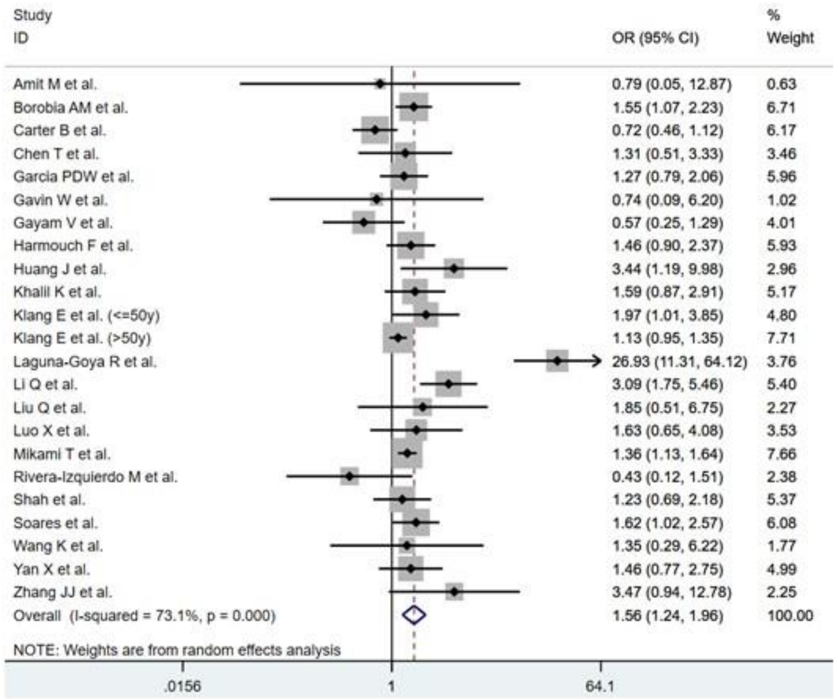

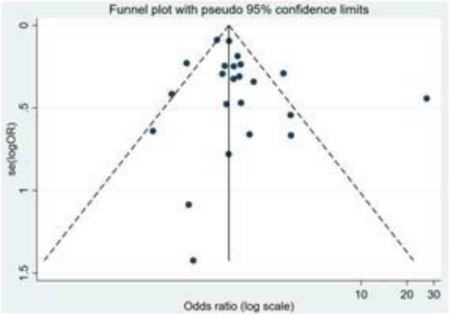

Egger's test: p-value=0.23

Unspecified comorbidity

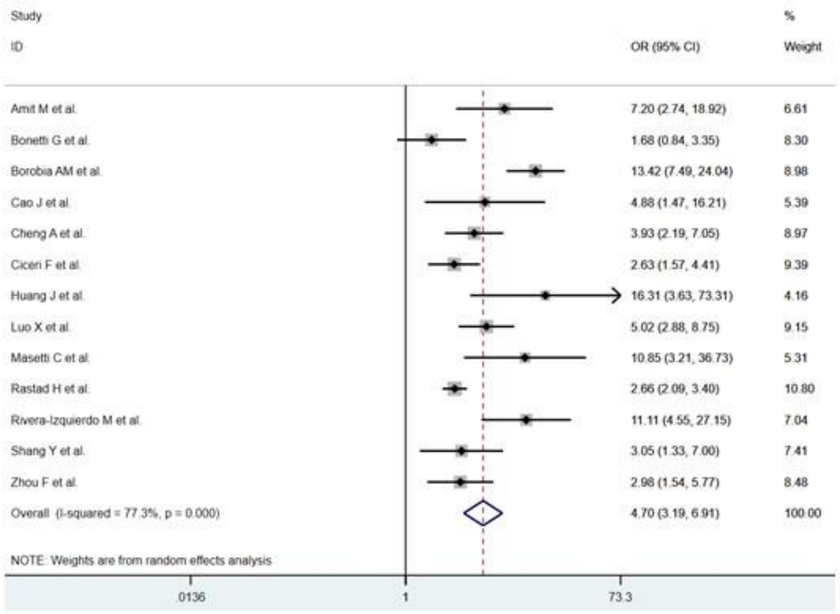

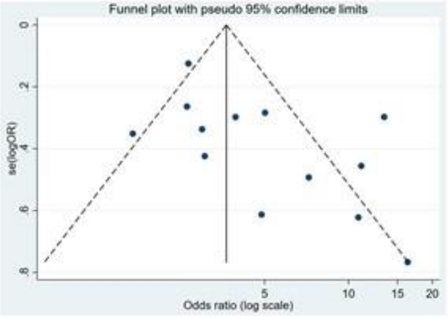

Egger's test: p-value=0.045

Stroke

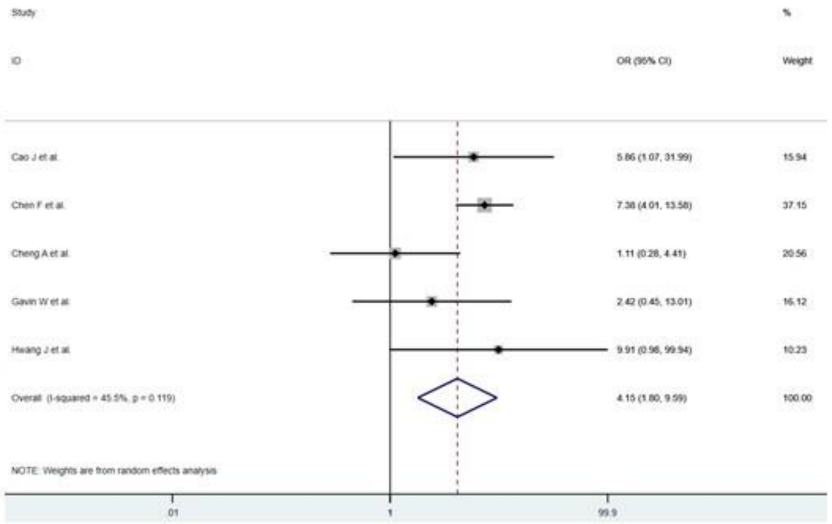

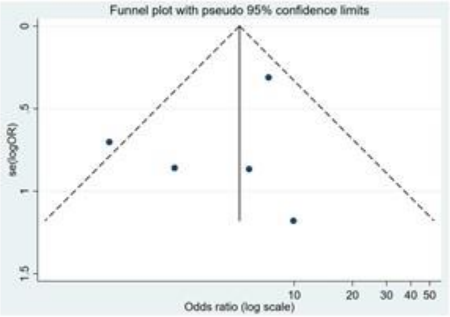

Egger's test: p-value=0.44

Kidney disease

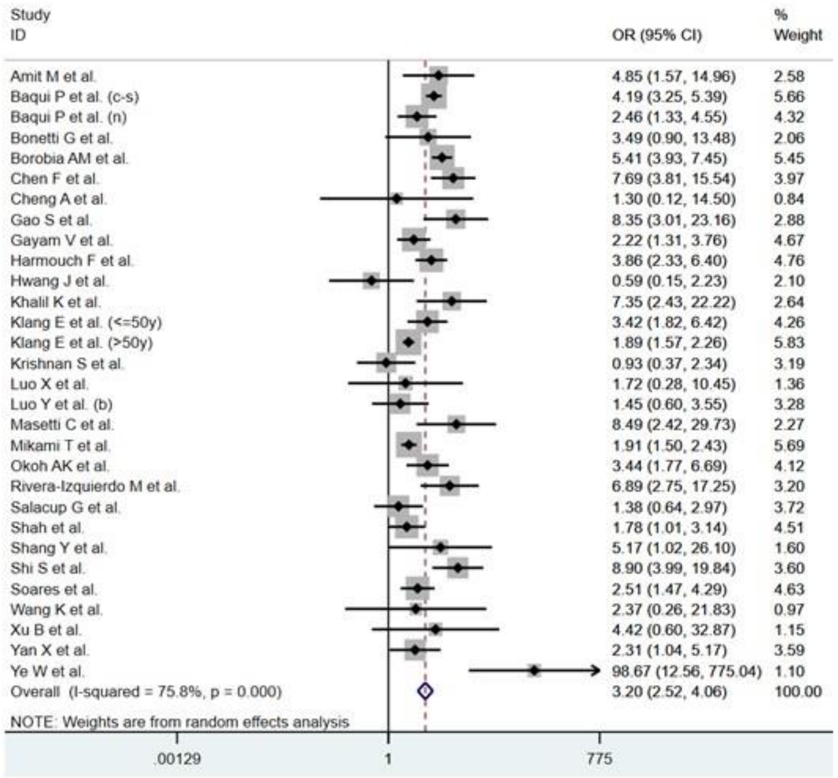

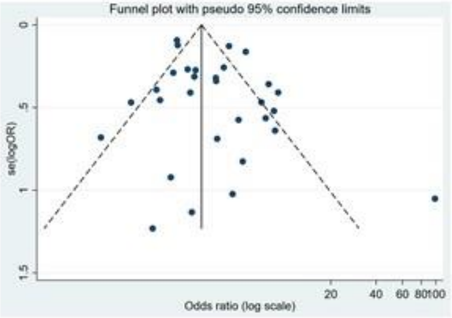

Egger's test: p-value=0.15

Cardiovascular disease

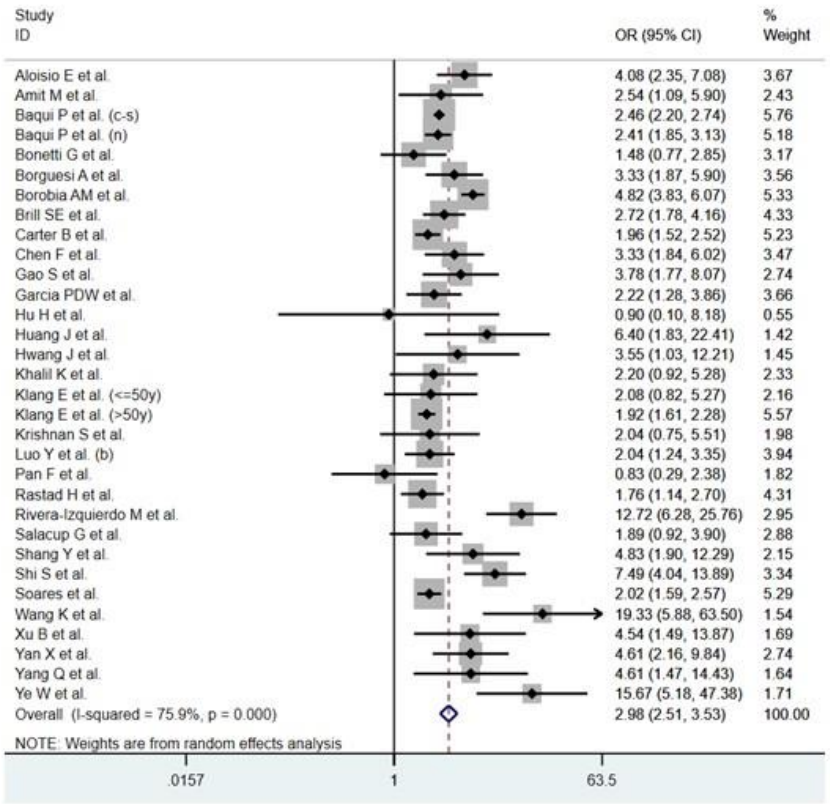

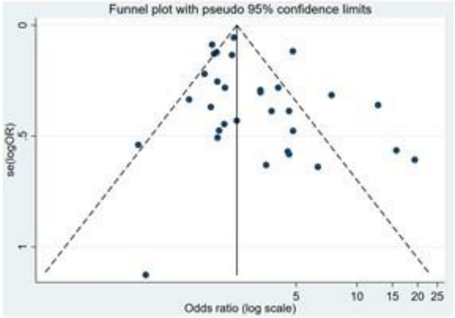

Egger's test: p-value=0.077

Hypertension

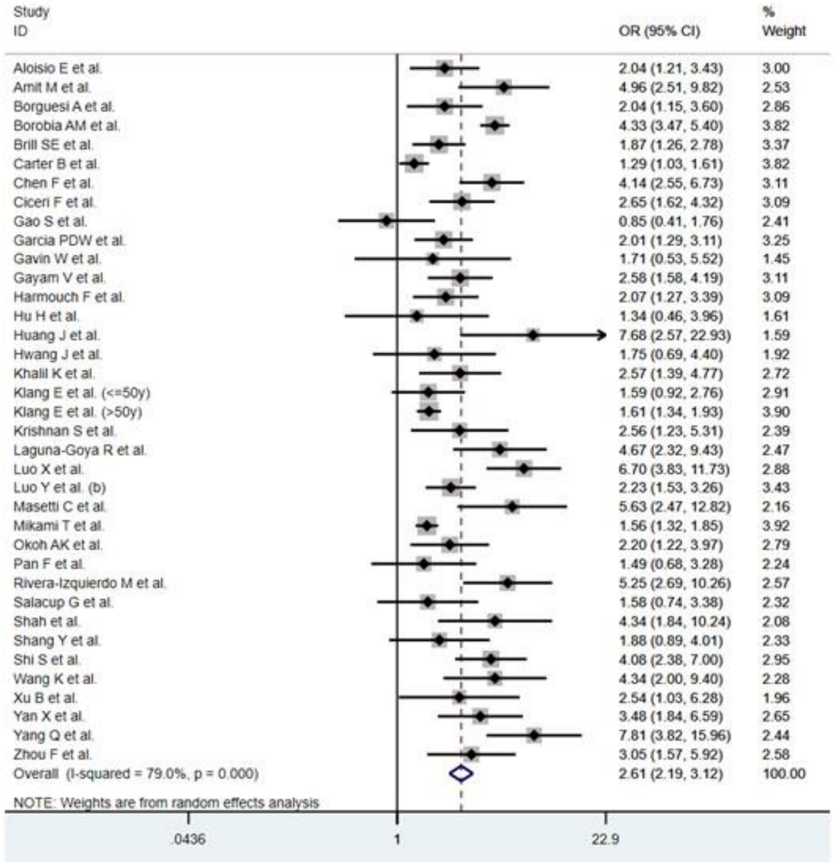

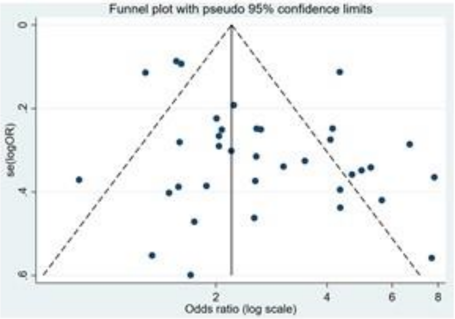

Egger's test: p-value=0.014

Malignancy

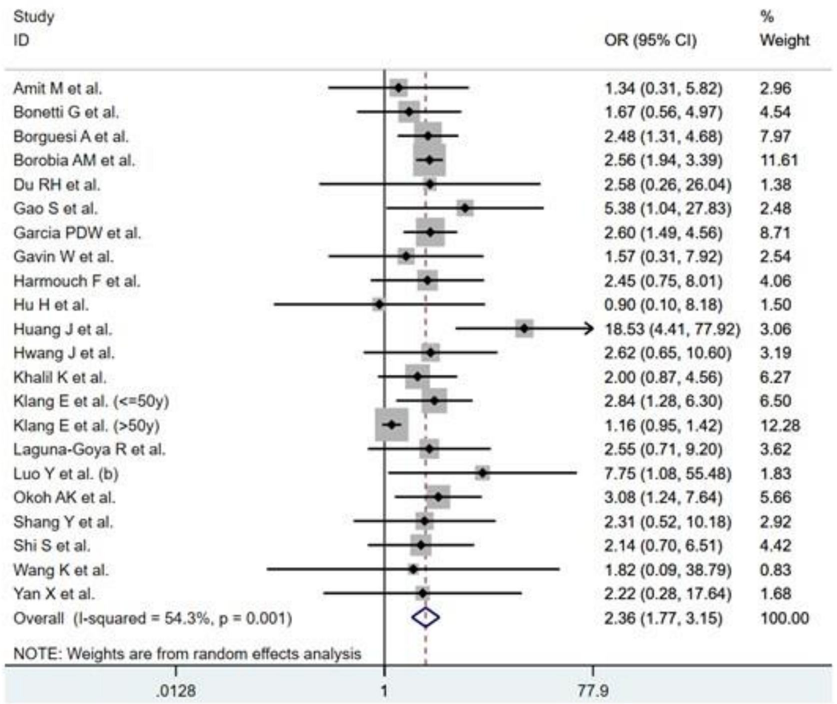

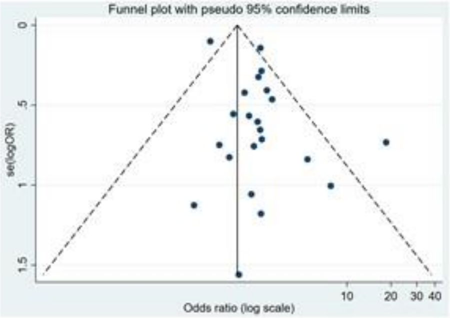

Egger's test: p-value=0.019

Diabetes

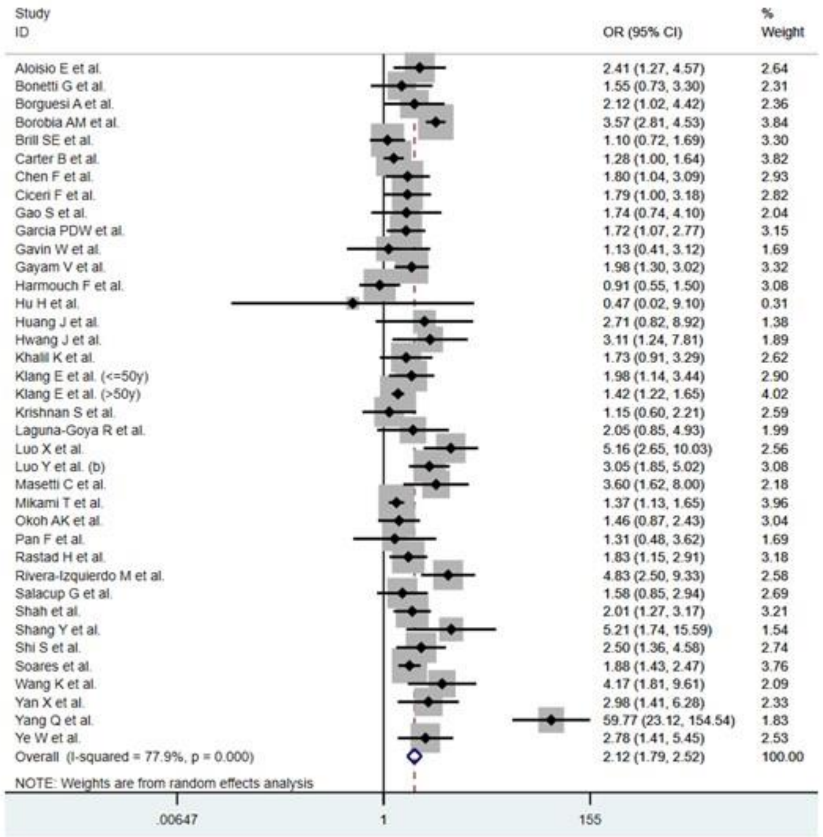

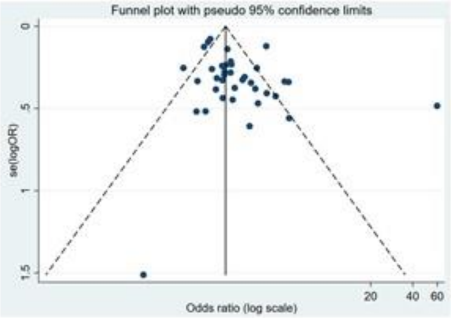

Egger's test: p-value=0.018

Pulmonary disease

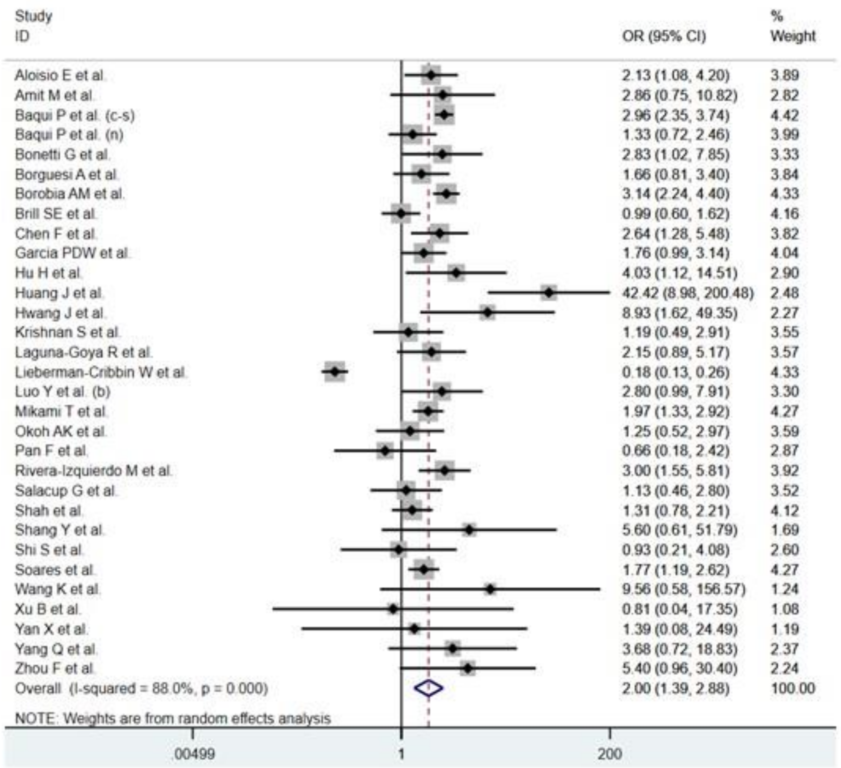

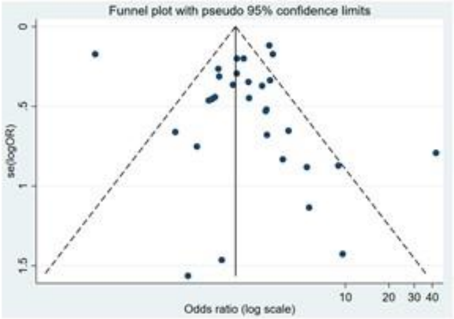

Egger's test: p-value=0.48

Liver disease

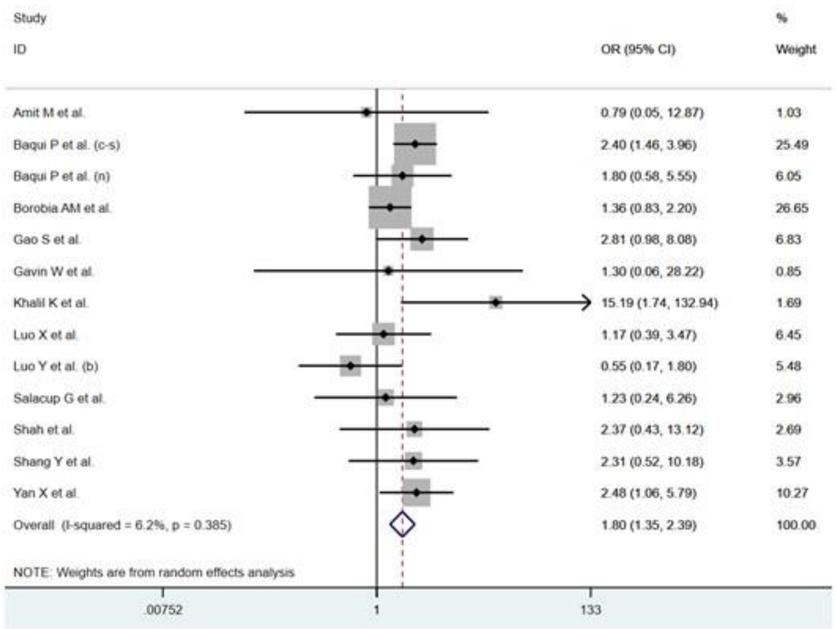

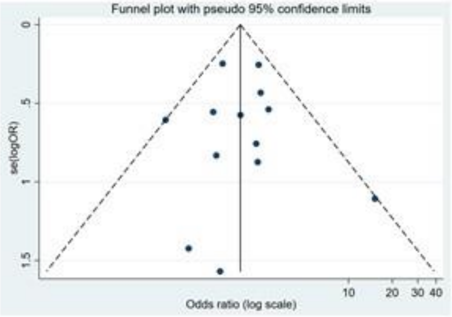

Egger’s test: p-value=0.92

Lymphocyte count

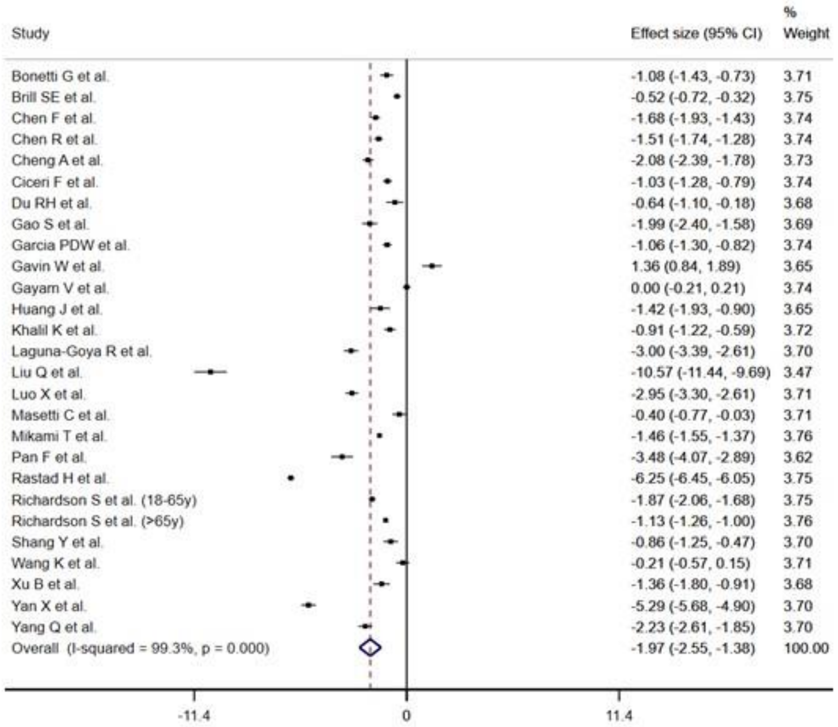

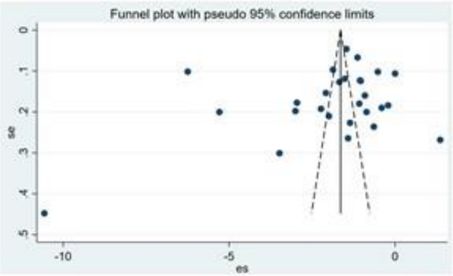

Egger's test: p-value=0.43

Leucocyte count

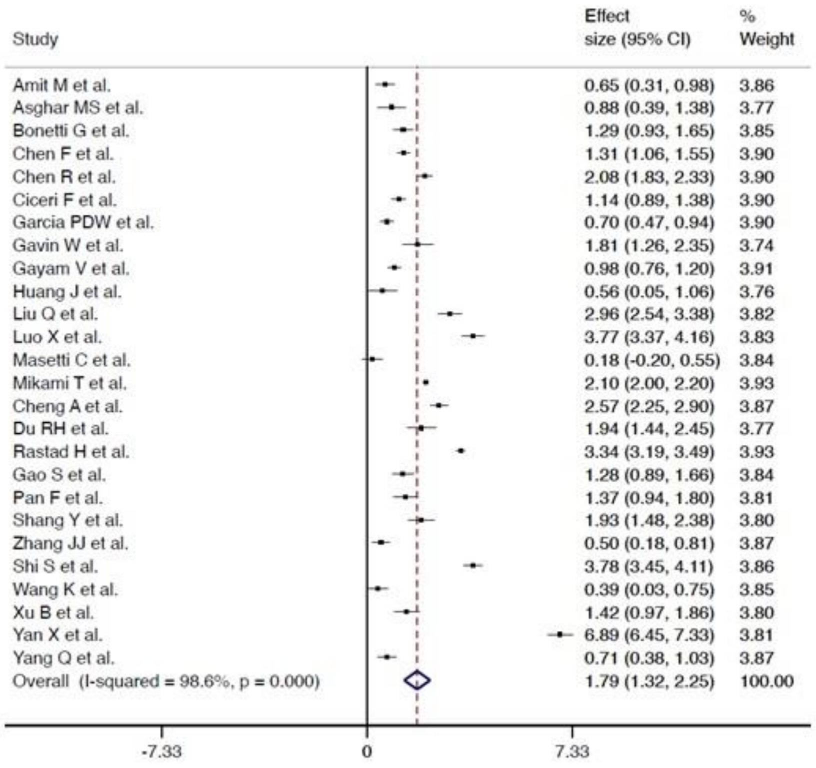

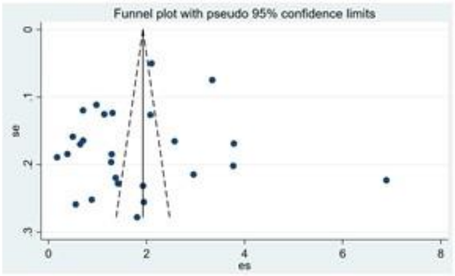

Egger's test: p-value=0.62

Neutrophil count

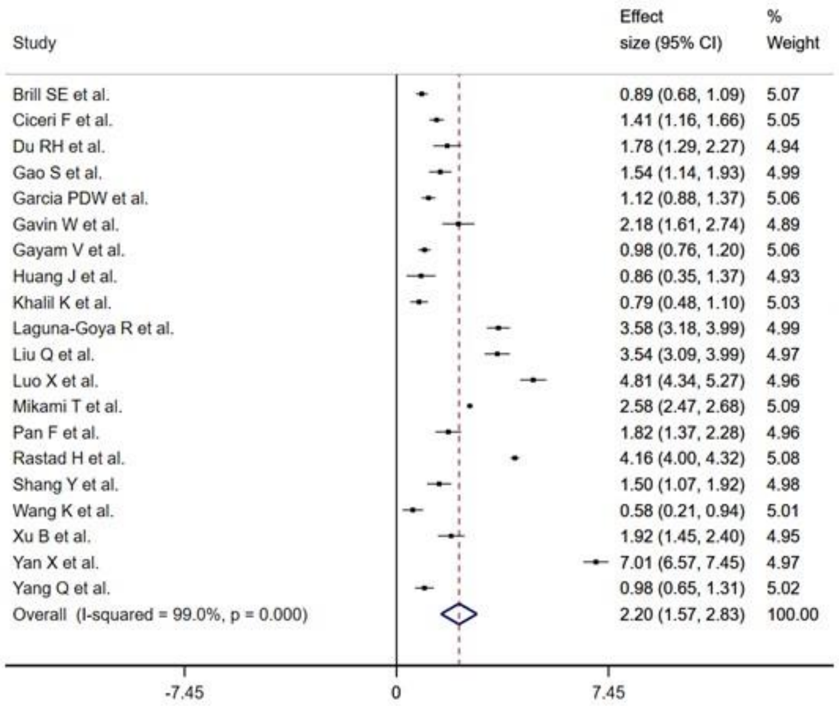

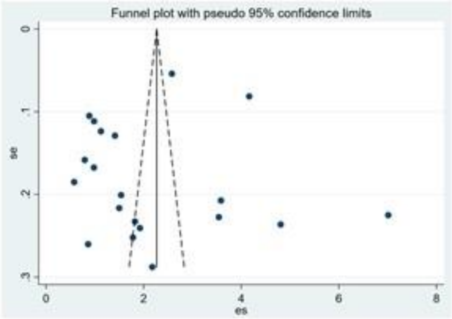

Egger’s test: p-value=0.48

Platelet count

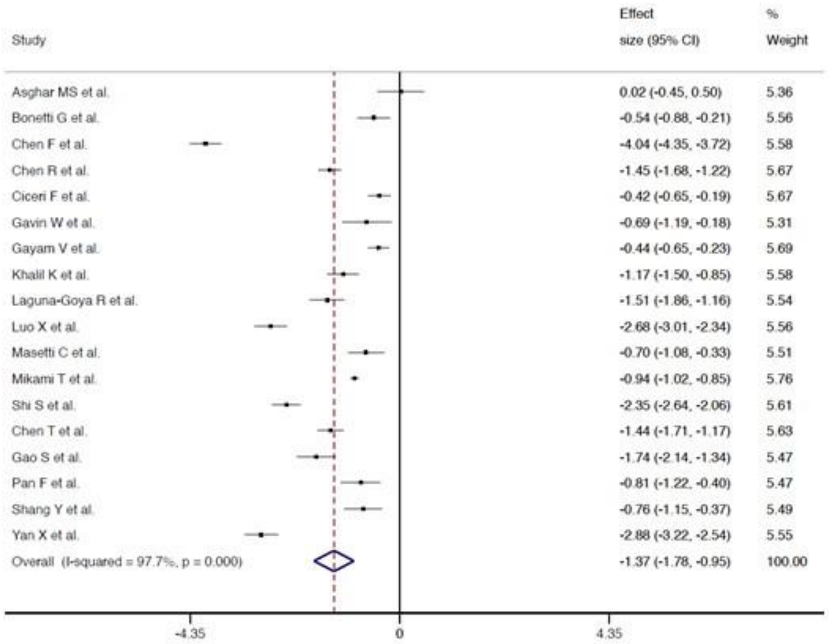

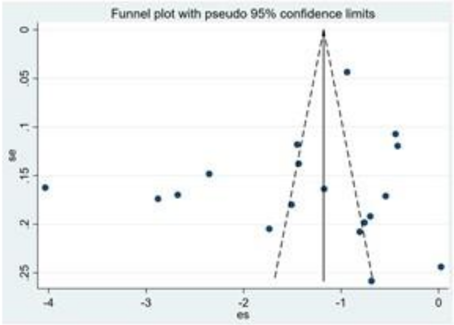

Egger's test: p-value=0.20

Hemoglobin

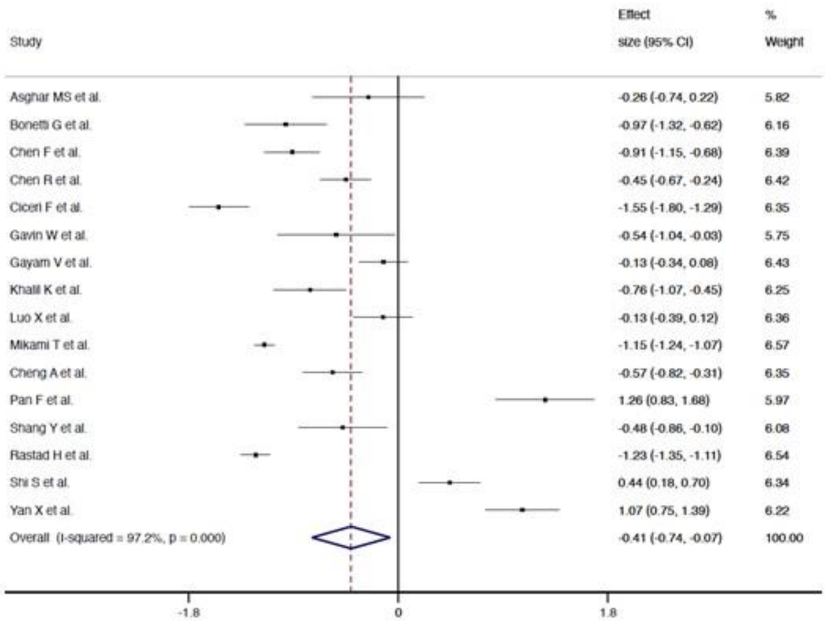

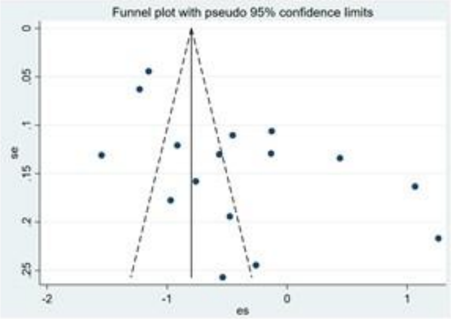

Egger's test: p-value=0.013

D-dimer

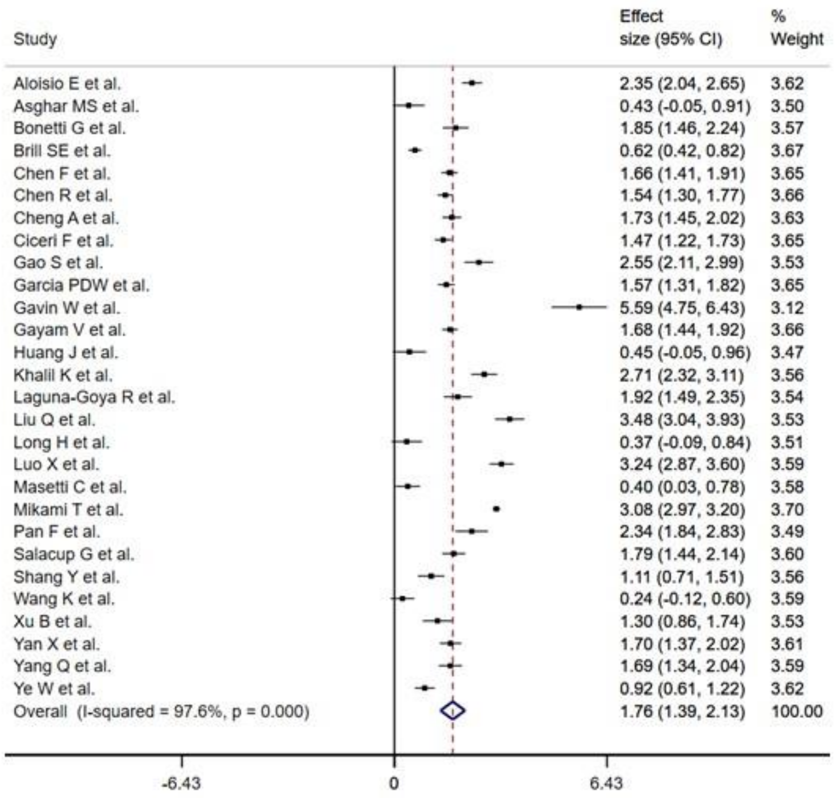

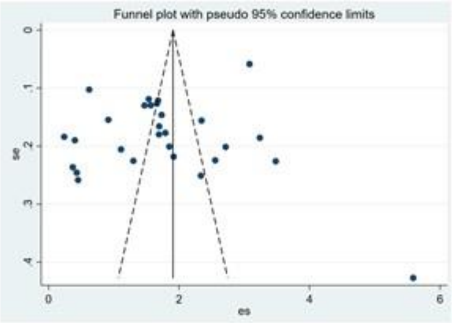

Egger's test: p-value=0.76

Prothrombin time

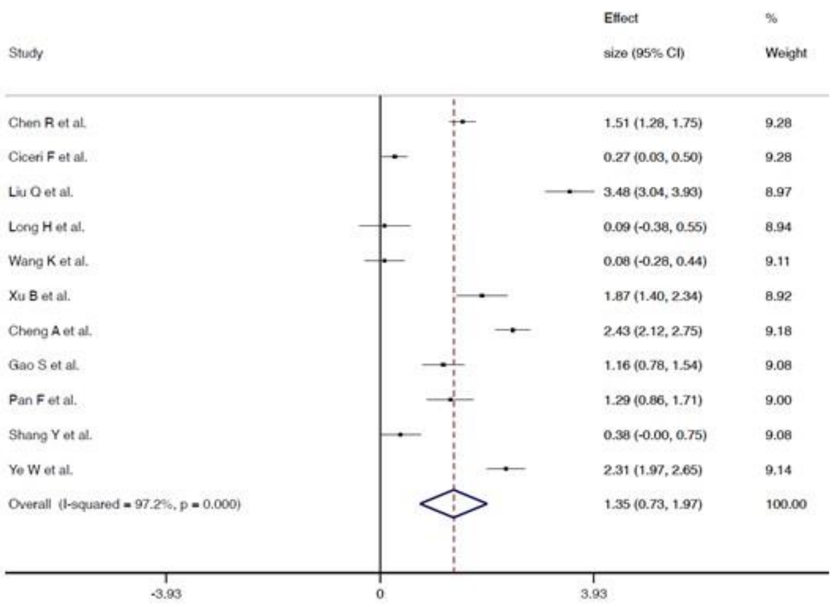

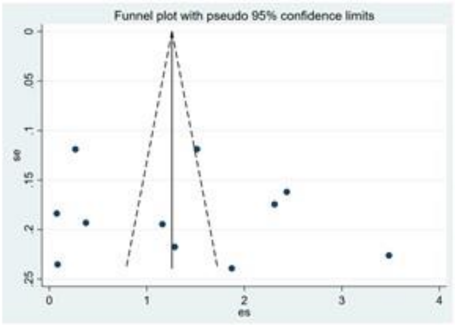

Egger’s test: p-value=0.081

APTT

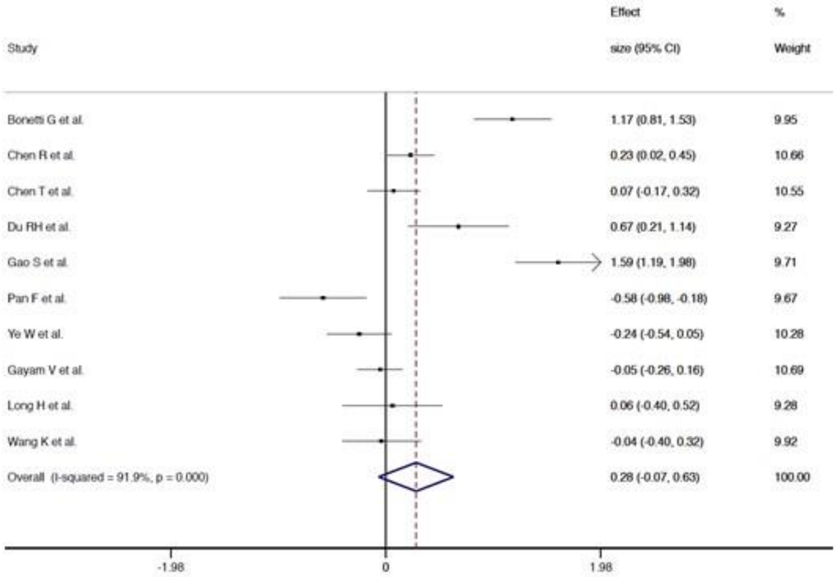

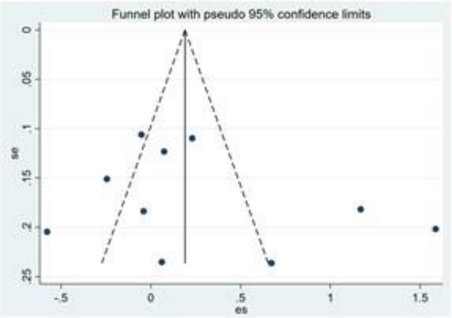

Egger's test: p-value=0.27

Albumin

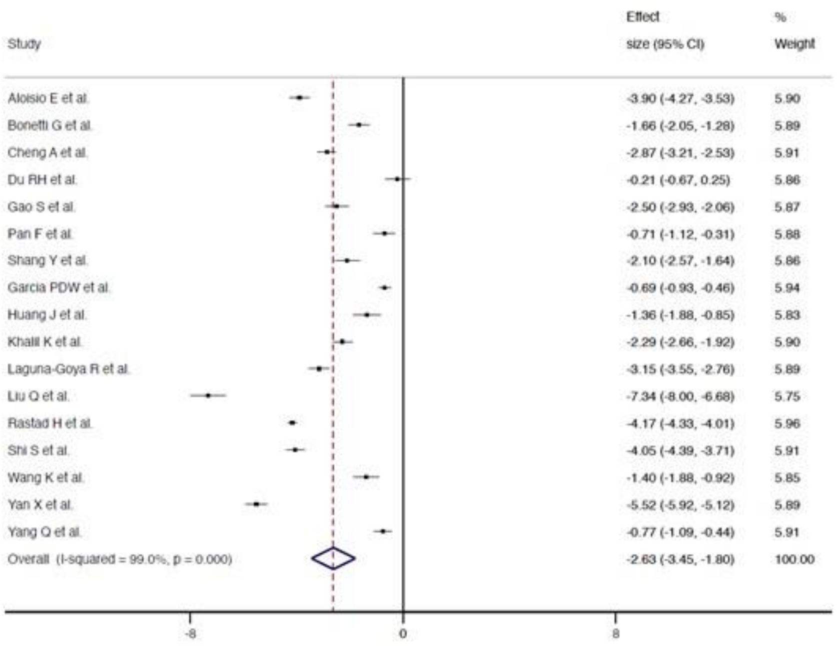

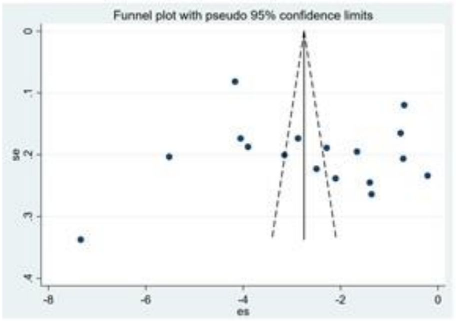

Egger's test: p-value=0.65

Total bilirubin

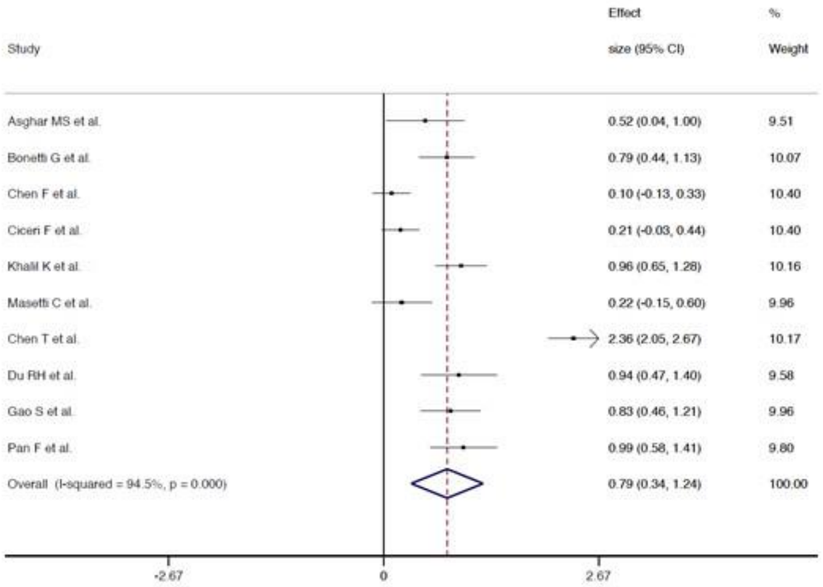

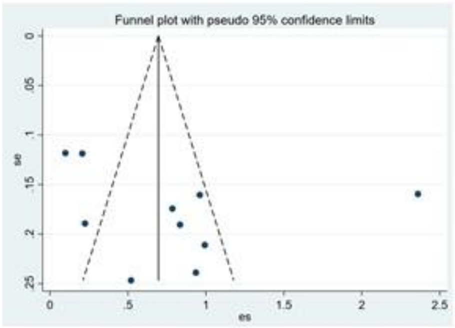

Egger's test: p-value=0.27

AST

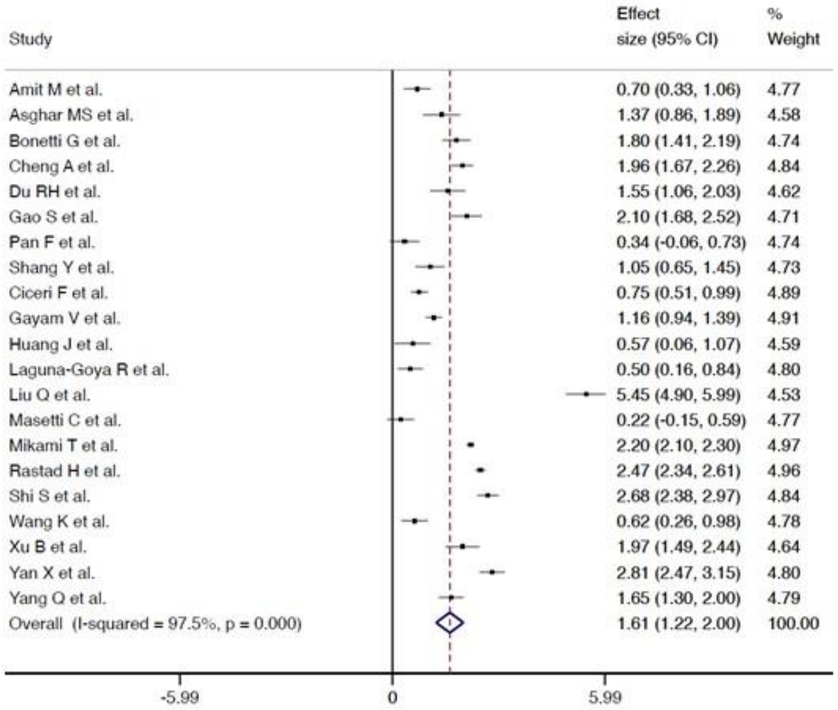

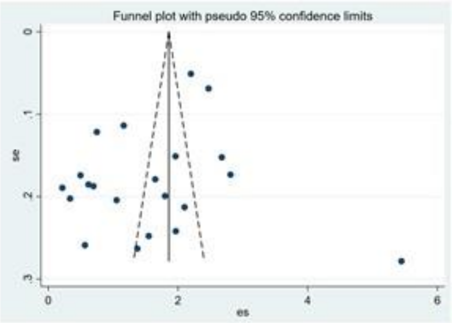

Egger's test: p-value=0.92

ALT

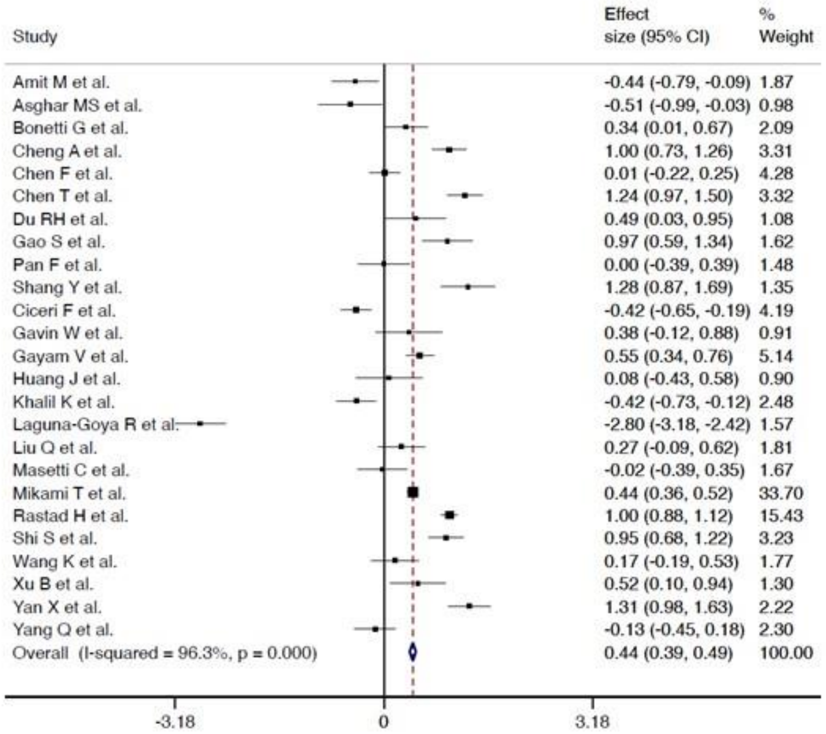

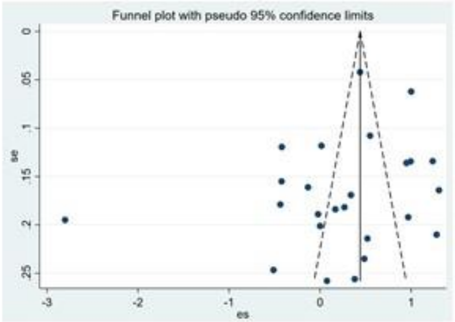

Egger's test: p-value=0.35

Urea nitrogen

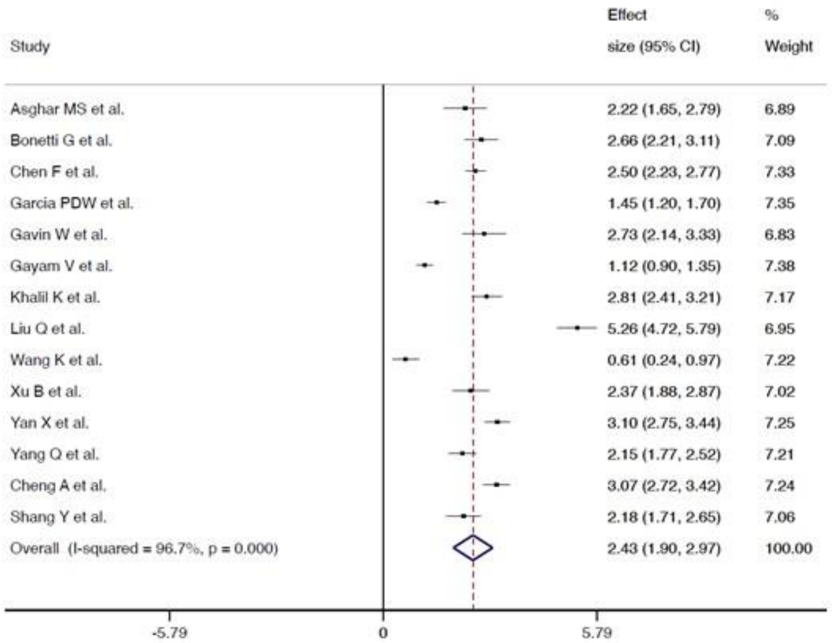

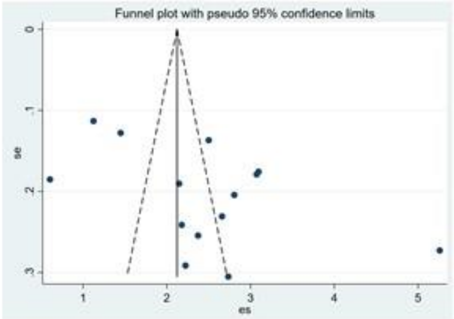

Egger's test: p-value=0.008

Creatinine

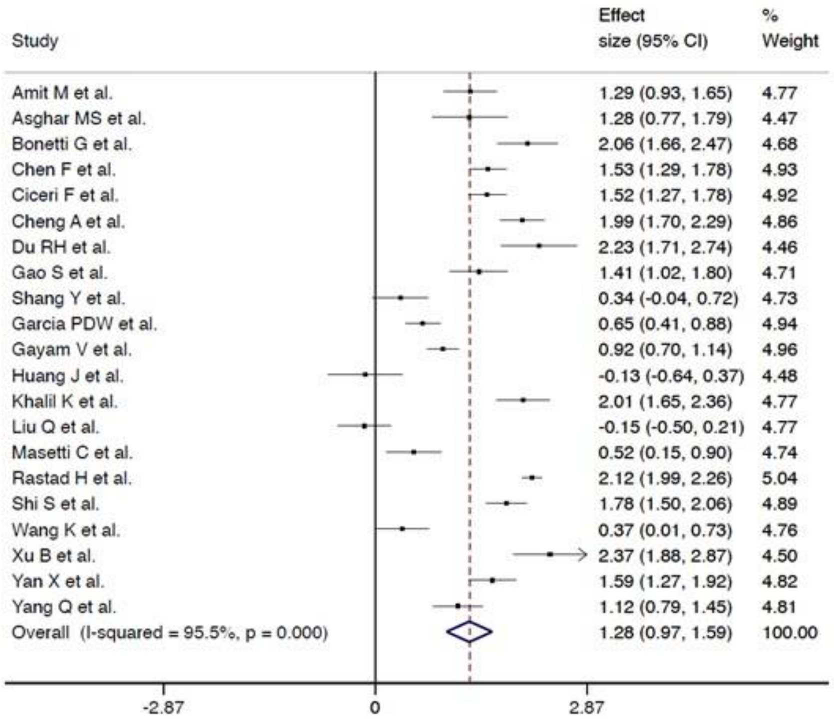

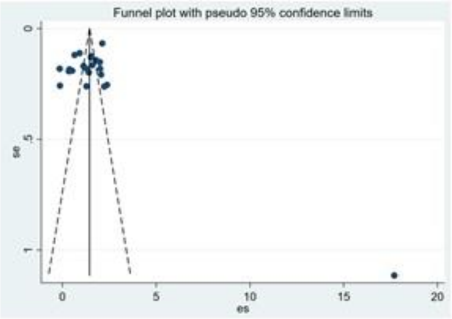

Egger's test: p-value=0.33

CRP

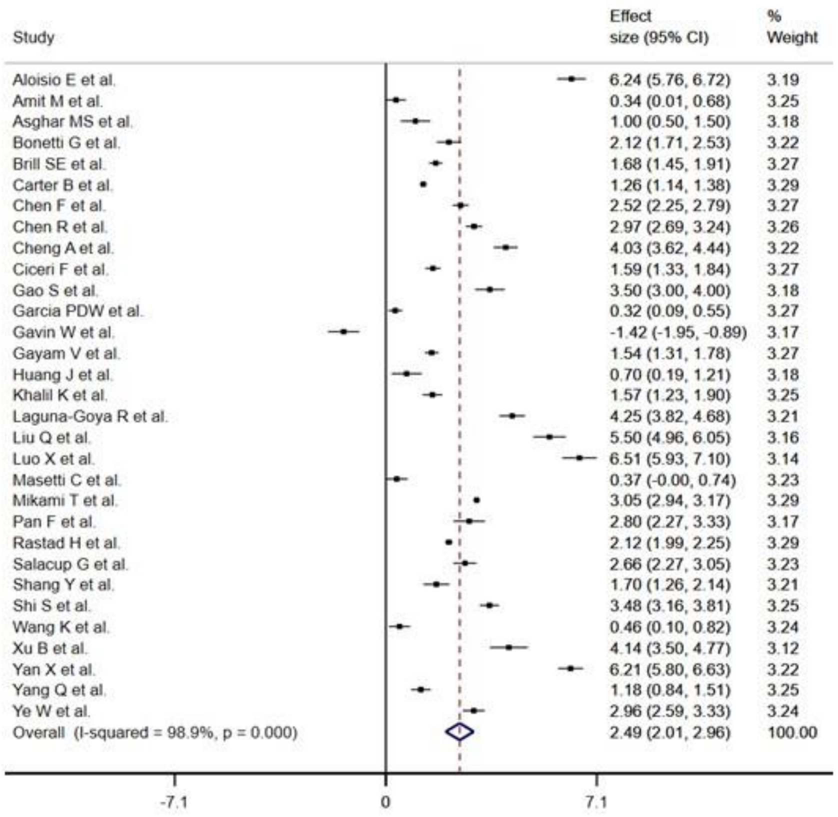

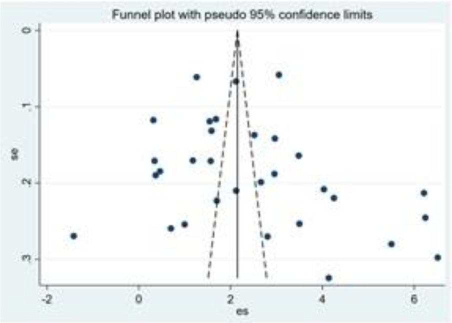

Egger's test: p-value=0.056

Interleukin-6

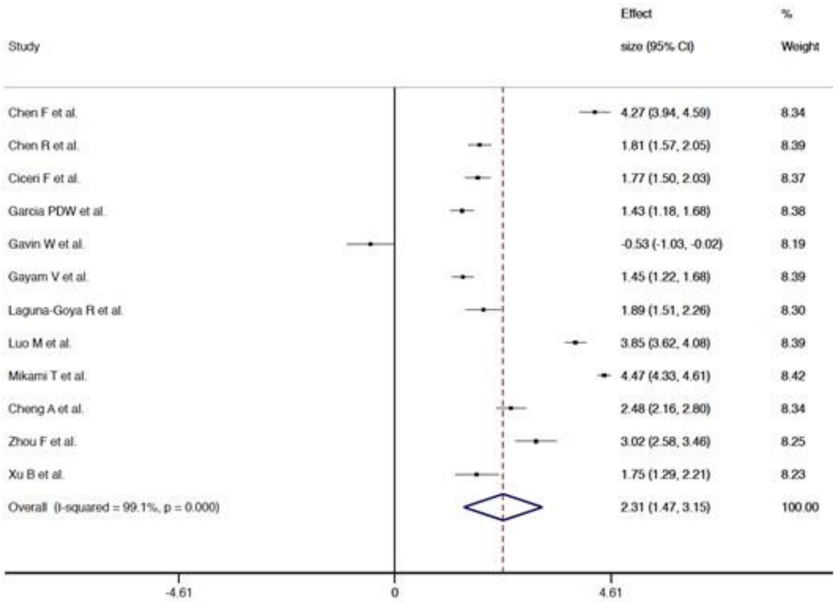

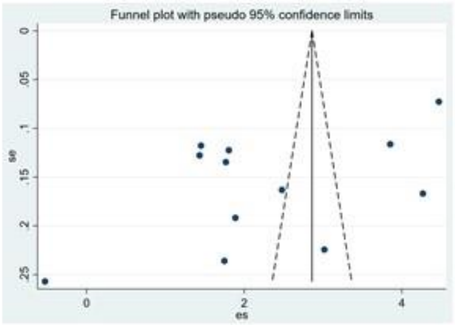

Egger’s test: p-value=0.63

LDH

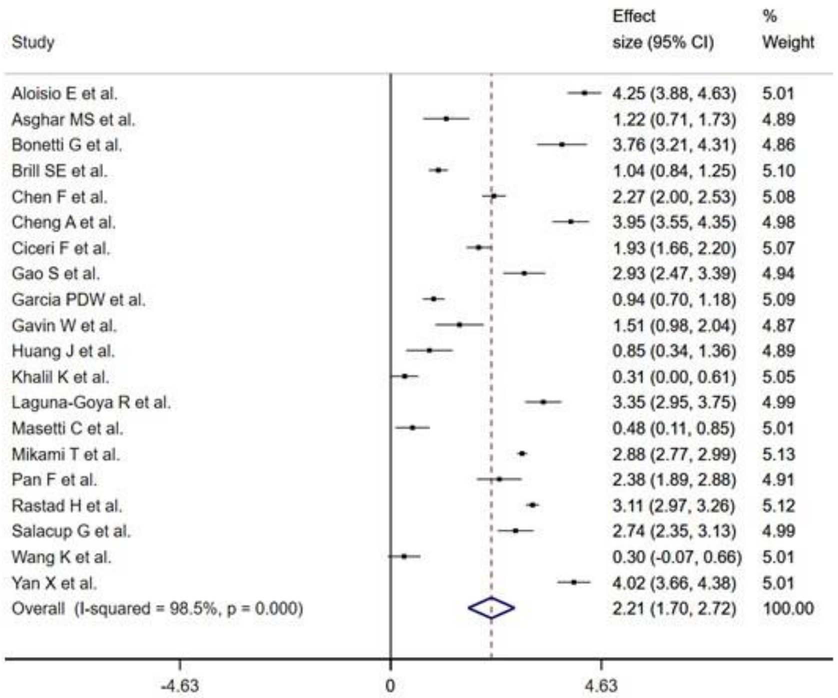

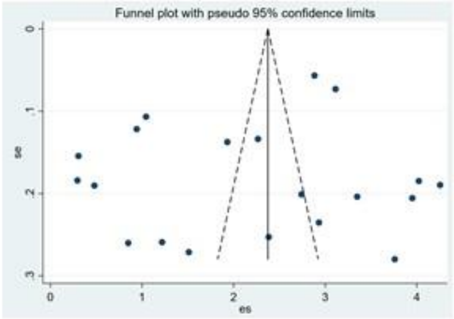

Egger's test: p-value=0.81

Procalcitonin

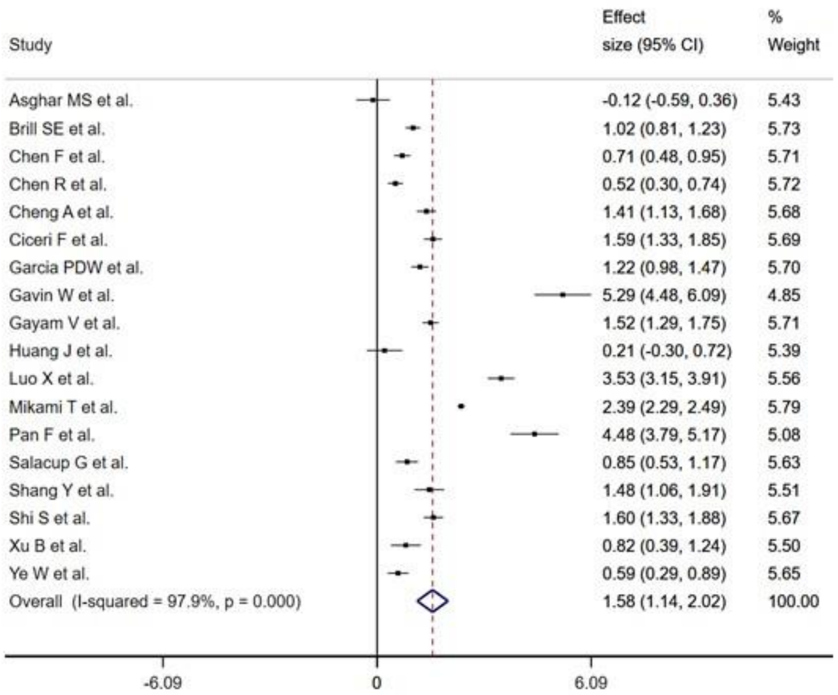

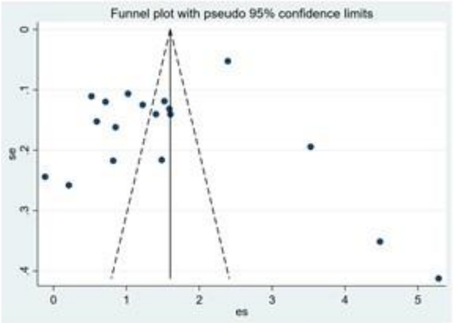

Egger’s test: p-value=0.37

Ferritin

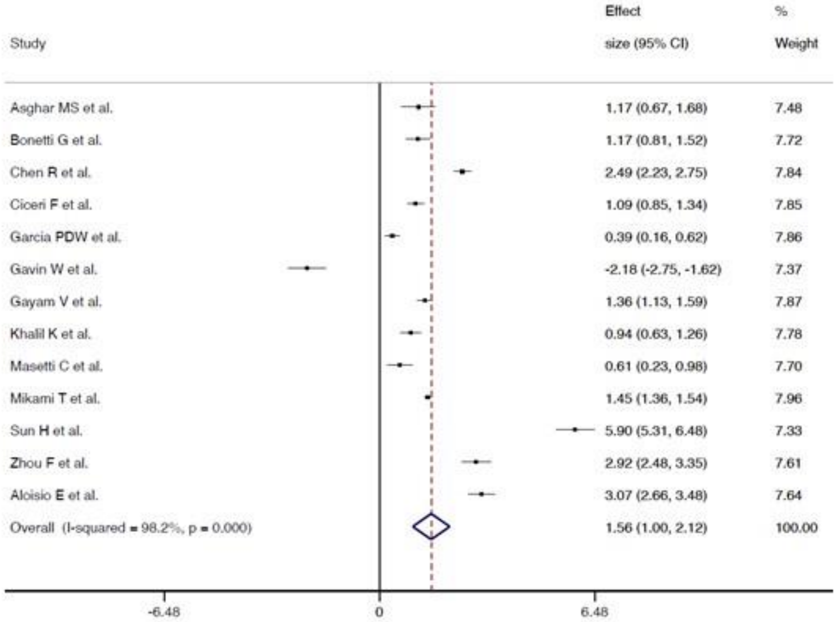

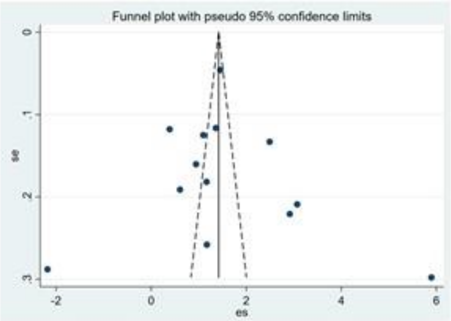

Egger's test: p-value=0.67

Cardiac troponin-I

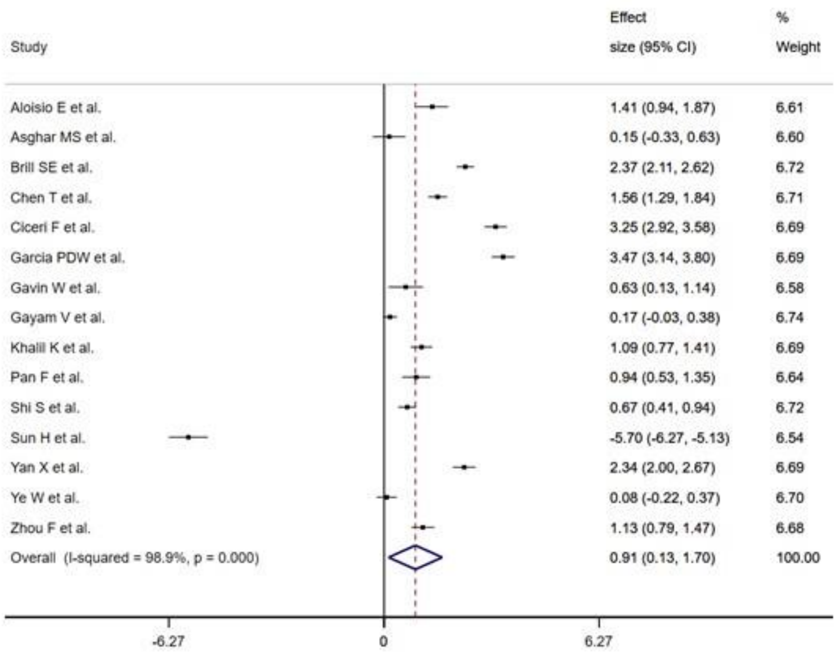

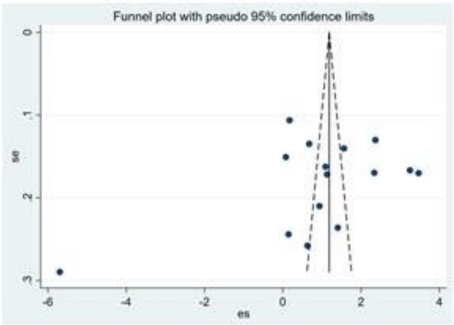

Egger's test: p-value=0.85

ESR

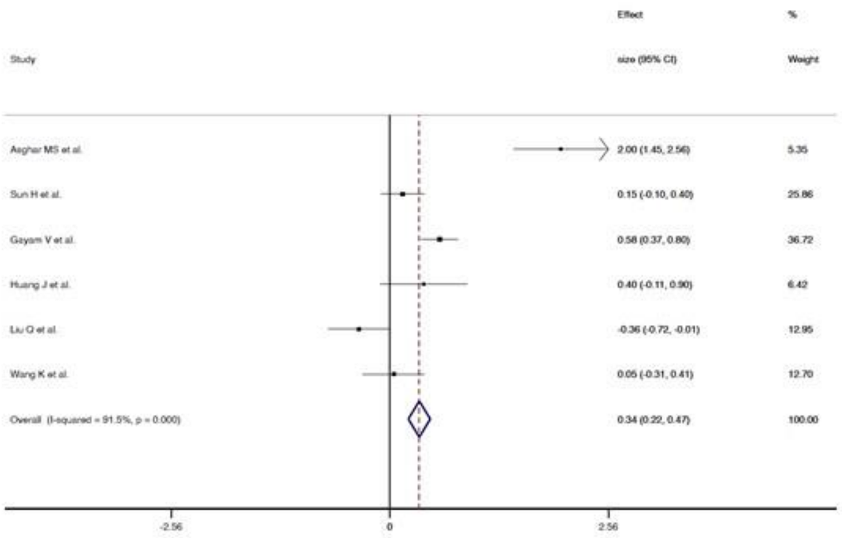

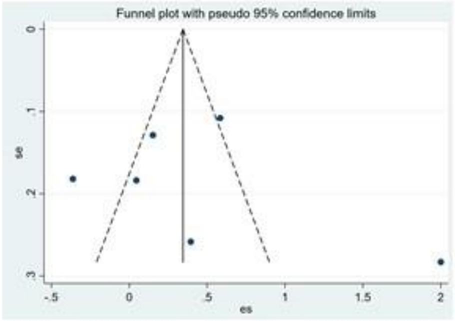

Egger’s test: p-value=0.80
