# Supplementary material for: Predictors of in-hospital COVID-19 mortality: A comprehensive systematic review and meta-analysis exploring differences by age, sex and health conditions
Source: PLoS One. 2020 Nov 3;15(11):e0241742. doi: 10.1371/journal.pone.0241742 (PMC7608886; doi:10.1371/journal.pone.0241742)
Supplement: S1 Table — (PDF) [file pone.0241742.s005.pdf]

Supporting Information

Predictors of in-hospital COVID-19 mortality: a comprehensive systematic review and meta-analysis exploring differences by age, sex and health conditions

S1 Table. Description of sub-groups and corresponding criteria.

Authors	Predominant age		Predominant sex		Predominant health condition		
	>60.0 years	Age (years)	≥60.0 % men	Male sex, %	>50.0 of prevalence	Most prevalent chronic condition of illness	%
Aloisio E et al.	Yes	61.3 ± 6.6	Yes	68.6	No	Hypertension	32.8
Amit M et al.	Yes	71.5 ± 6.3	Yes	69.0	Yes	Comorbidity	80.8
Asghar MS et al.	No	52.6 ± 15.7	Yes	69.0	No	-	
Baqui P et al. (Centra-South)	No	58.2 ± 17.8	No	58.0	No	Cardiovascular disease	34.6
Baqui P et al. (North)	No	58.8 ± 19.4	No	58.9	No	Cardiovascular disease	31.1
Bonetti G et al.	Yes	69.1 ± 8.9	Yes	66.7	Yes	Comorbidity	63.9
Borghesi A et al.	Yes	67.0 ± 5.8	Yes	64.2	Yes	Hypertension	51.0
Borobia AM et al.	Yes	61.5 ± 9.2	No	48.2	Yes	Comorbidity	78.5
Brill SE et al.	No	69.9 ± 7.0	No	59.8	No	Hypertension	43.2
Cao J et al.	No	53.0 ± 8.7	No	52.0	No	Comorbidity	46.1
Carter B et al.	Yes	73.0 ± 6.3	No	57.7	Yes	Hypertension	51.4
Chen F et al.	No	53.0 ± 9.8	No	44.7	No	Hypertension	34.8
Chen R et al.	No	56.0 ± 14.5	No	57.1	No	-	
Chen T et al.	No	59.5 ± 7.5	Yes	62.4	No	-	
Cheng A et al.	Yes	63.3 ± 5.5	Yes	60.0	Yes	Comorbidity	57.0

Ciceri F et al.	Yes	65.7 ± 7.4		Yes	71.8		Yes	Comorbidity	58.3
Deng Y et al.	No	55.1 ± 14.1		No	55.1		No	-	
Du RH et al.	No	57.6 ± 13.7		No	54.2		No	Malignancy	2.2
Gao S et al.	Yes	71.5 ± 2.9		No	48.0		Yes	Hypertension	54.8
Garcia PDW et al.	Yes	62.5 ± 5.2		Yes	75.1		Yes	"critically ill patients"	100.0
Gavin W et al.	No	60.0 ± 6.9		No	51.4		Yes	Hypertension	68.6
Gayam V et al.	Yes	66.5 ± 5.8		No	56.6		Yes	Hypertension	66.4
Harmouch F et al.	Yes	63.5 ± 21.2		No	57.1		Yes	Hypertension	50.3
Hu H et al.	Yes	60.8 ± 16.3		No	50.9		Yes	"critically ill patients"	100.0
Huang J et al.	No	53.4 ± 16.7		No	53.5		No	Comorbidity	33.1
Hwang JM et al.	Yes	67.6 ± 15.3		No	50.0		Yes	Hypertension	55.3
Khalil K et al.	Yes	66.9 ± 17.0		No	59.1		No	Hypertension	45.0
Klang E et al. (<=50y)	No	40.7 ± 3.9		Yes	69.4		No	Obesity	48.1
Klang E et al. (>50y)	Yes	71.1 ± 6.1		No	55.2		Yes	Hypertension	74.9
Krishnan S et al.	Yes	66.0 ± 13.0		Yes	62.5		Yes	Hypertension	73.0
Laguna-Goya R et al.	No	52.0 ± 4.6		Yes	63.3		No	Hypertension	27.7
Li Q et al.	No	55.5 ± 6.9		No	51.0		No	-	
Lieberman-Cribbin W et al.	-	NA		-	NA		No	Pulmonary disease	10.7
Liu Q et al.	Yes	62.5 ± 5.2		No	50.3		Yes	"critically ill patients"	100.0
Long H et al.	Yes	63.6 ± 13.9		No	57.4		Yes	"severe/critical patients"	66.1
Luo M et al.	No	60.0 ± 5.8		No	51.2		No	-	
Luo X et al.	No	55.8 ± 8.4		No	50.3		Yes	"severe/critical illness"	52.7
Luo Y et al. (a)	Yes	60.1 ± 15.2		No	49.4		No	-	
Luo Y et al. (b)	No	59.9 ± 15.3		No	50.5		No	Hypertension	27.6
Masetti C et al.	Yes	60.7 ± 14.2		Yes	64.6		Yes	Comorbidity	54.1
Mikami T et al.	Yes	65.5 ± 9.1		No	54.5		No	Hypertension	33.0
Okoh AK et al.	Yes	61.8 ± 7.2		No	51.0		Yes	Hypertension	69.7

Pan F et al.	Yes	66.9 ± 5.5		Yes	68.5		Yes	"severe COVID-19"	100.0
Rastad H et al.	No	54.8 ± 16.9		No	53.7		No	Comorbidity	25.3
Richardson S et al. (18-65y)	No	-		Yes	62.3		No	-	
Richardson S et al. (>65y)	Yes	-		No	57.1		No	-	
Rivera-Izquierdo M et al.	Yes	64.7 ± 15.4		No	55.0		Yes	Comorbidity	56.7
Ruan Q et al.	No	56.8 ± 15.0		Yes	68.0		No	-	
Salacup G et al.	Yes	66.5 ± 5.2		No	51.0		Yes	Hypertension	74.4
Shah P et al.	Yes	62.0 ± 6.3		No	41.8		Yes	Hypertension	79.7
Shang Y et al.	Yes	65.6 ± 4.8		Yes	64.6		Yes	Comorbidity	63.7
Shi S et al.	Yes	62.0 ± 6.3		No	48.0		Yes	"severe COVID-19"	100.0
Soares RCM et al.	-	NA		No	57.1		No	Cardiovascular disease	45.7
Sun H et al.	Yes	69.7 ± 3.7		No	54.5		No	-	
Wang K et al.	No	48.3 ± 15.4		No	48.2		No	Hypertension	15.6
Xu B et al.	Yes	60.9 ± 6.5		No	52.4		No	Hypertension	21.4
Yan X et al.	Yes	61.3 ± 6.0		No	49.1		No	Hypertension	23.4
Yang Q et al.	No	53.9 ± 17.1		No	50.0		No	Hypertension	37.2
Yang X et al.	No	57.6 ± 6.8		No	52.6		No	-	
Ye W et al.	No	53.5 ± 13.8		No	49.6		No	Diabetes	16.3
Yu C et al.	Yes	62.5 ± 5.8		No	50.3		No	-	
Zhang JJ et al.	No	56.0 ± 19.1		No	53.3		No	-	
Zhou F et al	No	56.3 ± 6.1		Yes	62.0		No	Hypertension	47.6

NA: not available.
