# Supplementary material for: Predictors of in-hospital COVID-19 mortality: A comprehensive systematic review and meta-analysis exploring differences by age, sex and health conditions
Source: PLoS One. 2020 Nov 3;15(11):e0241742. doi: 10.1371/journal.pone.0241742 (PMC7608886; doi:10.1371/journal.pone.0241742)
Supplement: S2 Table — (PDF) [file pone.0241742.s006.pdf]

Supporting Information

Predictors of in-hospital COVID-19 mortality: a comprehensive systematic review and meta-analysis exploring differences by age, sex and health conditions

S2 Table. Risk of bias assessment of the included studies[a].

Authors	Domain					
	Study Participation	Study Attrition	Prognostic Factor Measurement	Outcome Measurement	Study Confounding	Statistical Analysis and Reporting
Aloisio E et al.	Low risk of bias	Low risk of bias	Moderate risk of bias	Low risk of bias	Moderate risk of bias	Low risk of bias
Amit M et al.	Low risk of bias	Low risk of bias	Low risk of bias	Low risk of bias	Low risk of bias	Low risk of bias
Asghar MS et al.	Low risk of bias	Low risk of bias	Low risk of bias	Moderate risk of bias	Moderate risk of bias	Low risk of bias
Baqui P et al.	Low risk of bias	Low risk of bias	Moderate risk of bias	Moderate risk of bias	Low risk of bias	Low risk of bias
Bonetti G et al.	Low risk of bias	Low risk of bias	Moderate risk of bias	Low risk of bias	Low risk of bias	Low risk of bias
Borguesi A et al.	Low risk of bias	Low risk of bias	Low risk of bias	Moderate risk of bias	Low risk of bias	Low risk of bias
Borobia AM et al.	Low risk of bias	Low risk of bias	Moderate risk of bias	Moderate risk of bias	Low risk of bias	Low risk of bias
Brill SE et al.	Low risk of bias	Low risk of bias	Low risk of bias	Low risk of bias	Moderate risk of bias	Low risk of bias
Cao J et al.	Low risk of bias	Low risk of bias	Low risk of bias	Low risk of bias	Moderate risk of bias	Low risk of bias
Carter B et al.	Low risk of bias	Low risk of bias	Low risk of bias	Low risk of bias	Low risk of bias	Low risk of bias
Chen F et al.	Low risk of bias	Low risk of bias	Moderate risk of bias	Moderate risk of bias	Low risk of bias	Low risk of bias
Chen R et al.	Low risk of bias	Low risk of bias	Low risk of bias	Moderate risk of bias	Low risk of bias	Low risk of bias
Chen T et al.	Low risk of bias	Low risk of bias	Low risk of bias	Low risk of bias	Moderate risk of bias	Low risk of bias
Cheng A et al	Low risk of bias	Low risk of bias	Low risk of bias	Low risk of bias	Low risk of bias	Low risk of bias
Ciceri F et al.	Low risk of bias	Low risk of bias	Low risk of bias	Low risk of bias	Low risk of bias	Low risk of bias

Deng Y et al.	Low risk of bias	Low risk of bias	Low risk of bias	Moderate risk of bias	Moderate risk of bias	Low risk of bias
Du RH et al.	Low risk of bias	Low risk of bias	Low risk of bias	Moderate risk of bias	Low risk of bias	Low risk of bias
Gao S et al.	Low risk of bias	Low risk of bias	Low risk of bias	Low risk of bias	Moderate risk of bias	Low risk of bias
Garcia PDW et al.	Low risk of bias	Low risk of bias	Low risk of bias	Low risk of bias	Low risk of bias	Low risk of bias
Gavin W et al.	Low risk of bias	Low risk of bias	Moderate risk of bias	Moderate risk of bias	Moderate risk of bias	Low risk of bias
Gayam V et al.	Low risk of bias	Low risk of bias	Low risk of bias	Moderate risk of bias	Low risk of bias	Low risk of bias
Harmouch F et al.	Low risk of bias	Low risk of bias	Moderate risk of bias	Moderate risk of bias	Low risk of bias	Low risk of bias
Hu H et al.	Low risk of bias	Low risk of bias	Low risk of bias	Moderate risk of bias	Low risk of bias	Low risk of bias
Huang J et al.	Low risk of bias	Low risk of bias	Low risk of bias	Low risk of bias	Low risk of bias	Low risk of bias
Hwang JM et al.	Low risk of bias	Low risk of bias	Low risk of bias	Moderate risk of bias	Low risk of bias	Low risk of bias
Khalil K et al.	Low risk of bias	Low risk of bias	Low risk of bias	Moderate risk of bias	Low risk of bias	Low risk of bias
Klang E et al.	Low risk of bias	Low risk of bias	Low risk of bias	Low risk of bias	Low risk of bias	Low risk of bias
Krishnan S et al.	Low risk of bias	Low risk of bias	Low risk of bias	Moderate risk of bias	Moderate risk of bias	Low risk of bias
Laguna-Goya R et al.	Low risk of bias	Low risk of bias	Low risk of bias	Moderate risk of bias	Low risk of bias	Low risk of bias
Li Q et al.	Low risk of bias	Low risk of bias	Low risk of bias	Low risk of bias	Low risk of bias	Low risk of bias
Lieberman-Cribbin W et al.	Low risk of bias	Low risk of bias	Low risk of bias	Moderate risk of bias	Low risk of bias	Low risk of bias
Liu Q et al.	Low risk of bias	Low risk of bias	Low risk of bias	Moderate risk of bias	Low risk of bias	Low risk of bias
Long H et al.	Low risk of bias	Low risk of bias	Low risk of bias	Low risk of bias	Moderate risk of bias	Low risk of bias
Luo M et al.	Low risk of bias	Low risk of bias	Low risk of bias	Low risk of bias	Low risk of bias	Low risk of bias
Luo X et al.	Low risk of bias	Low risk of bias	Low risk of bias	Moderate risk of bias	Low risk of bias	Low risk of bias
Luo Y et al. (a)	Low risk of bias	Low risk of bias	Low risk of bias	Low risk of bias	Low risk of bias	Low risk of bias
Luo Y et al. (b)	Low risk of bias	Low risk of bias	Low risk of bias	Moderate risk of bias	Low risk of bias	Low risk of bias
Masetti C et al.	Low risk of bias	Low risk of bias	Low risk of bias	Low risk of bias	Low risk of bias	Low risk of bias
Mikami T et al.	Low risk of bias	Low risk of bias	Low risk of bias	Low risk of bias	Low risk of bias	Low risk of bias
Okoh AK et al.	Low risk of bias	Low risk of bias	Low risk of bias	Low risk of bias	Low risk of bias	Low risk of bias
Pan F et al.	Low risk of bias	Low risk of bias	Low risk of bias	Low risk of bias	Low risk of bias	Low risk of bias
Rastad H et al.	Low risk of bias	Low risk of bias	Low risk of bias	Low risk of bias	Low risk of bias	Low risk of bias

Richardson S et al.	Low risk of bias	Low risk of bias	Low risk of bias	Moderate risk of bias	Moderate risk of bias	Low risk of bias
Rivera-Izquierdo M et al.	Low risk of bias	Low risk of bias	Low risk of bias	Moderate risk of bias	Low risk of bias	Low risk of bias
Ruan Q et al.	Low risk of bias	Low risk of bias	Low risk of bias	Low risk of bias	Moderate risk of bias	Low risk of bias
Salacup G et al.	Low risk of bias	Low risk of bias	Low risk of bias	Moderate risk of bias	Low risk of bias	Low risk of bias
Shah P et al.	Low risk of bias	Low risk of bias	Low risk of bias	Moderate risk of bias	Low risk of bias	Low risk of bias
Shang Y et al.	Low risk of bias	Low risk of bias	Low risk of bias	Moderate risk of bias	Low risk of bias	Low risk of bias
Shi S et al.	Low risk of bias	Low risk of bias	Low risk of bias	Low risk of bias	Low risk of bias	Low risk of bias
Soares RCM et al.	Low risk of bias	Low risk of bias	Low risk of bias	Moderate risk of bias	Low risk of bias	Low risk of bias
Sun H et al.	Low risk of bias	Low risk of bias	Low risk of bias	Low risk of bias	Low risk of bias	Low risk of bias
Wang K et al.	Low risk of bias	Low risk of bias	Low risk of bias	Low risk of bias	Low risk of bias	Low risk of bias
Xu B et al.	Low risk of bias	Low risk of bias	Low risk of bias	Moderate risk of bias	Low risk of bias	Low risk of bias
Yan X et al.	Low risk of bias	Low risk of bias	Low risk of bias	Moderate risk of bias	Low risk of bias	Low risk of bias
Yang Q et al.	Low risk of bias	Low risk of bias	Low risk of bias	Moderate risk of bias	Low risk of bias	Low risk of bias
Yang X et al.	Low risk of bias	Low risk of bias	Moderate risk of bias	Moderate risk of bias	Moderate risk of bias	Low risk of bias
Ye W et al.	Low risk of bias	Low risk of bias	Low risk of bias	Moderate risk of bias	Low risk of bias	Low risk of bias
Yu C et al.	Low risk of bias	Low risk of bias	Low risk of bias	Moderate risk of bias	Low risk of bias	Low risk of bias
Zhang JJ et al.	Low risk of bias	Low risk of bias	Low risk of bias	Moderate risk of bias	Low risk of bias	Low risk of bias
Zhou F et al.	Low risk of bias	Low risk of bias	Low risk of bias	Moderate risk of bias	Low risk of bias	Low risk of bias

[a] Quality In Prognosis Studies (QUIPS) tool to assess risk of bias in studies of prognostic factors (Hayden JA et al. Assessing Bias in Studies of Prognostic Factors. Ann Intern Med. 2013;158:280-286).
