# Supplementary material for: Predictors of in-hospital COVID-19 mortality: A comprehensive systematic review and meta-analysis exploring differences by age, sex and health conditions
Source: PLoS One. 2020 Nov 3;15(11):e0241742. doi: 10.1371/journal.pone.0241742 (PMC7608886; doi:10.1371/journal.pone.0241742)
Supplement: S3 Table — (PDF) [file pone.0241742.s007.pdf]

Supporting Information

Predictors of in-hospital COVID-19 mortality: a comprehensive systematic review and meta-analysis exploring differences by age, sex and health conditions

S3 Table. Main meta-analyses results obtained in sensitivity analyses excluding studies with moderate risk of bias in more than one domain[a].

Potential predictor	Pooled-OR (95% CI)	Potential predictor	Pooled-ES (95% CI)
Dyspnoea	2.54 (1.84, 3.50)*	BMI	0.22 (-0.28, 0.71)
Fatigue	1.30 (0.59, 2.87)	Lymphocyte count	-2.11 (-2.76, -1.46)*
Fever	0.77 (0.64, 0.94)*	Leucocyte count	2.90 (2.08, 3.71)*
Myalgia	0.77 (0.32, 1.82)	Neutrophil count	3.37 (2.50, 4.25)*
Cough	0.75 (0.61, 0.93)*	Platelet count	-1.45 (-1.89, -1.02)*
Vomiting	0.60 (0.40, 0.89)*	Hemoglobin	-0.37 (-0.83, 0.10)
Diarrhoea	0.59 (0.43, 0.81)*	D-dimer	2.81 (2.30, 3.31)*
Headache	0.50 (0.28, 0.91)*	Prothrombin time	2.13 (1.17, 3.08)*
Obesity	0.99 (0.74, 1.33)	APTT	0.37 (-0.07, 0.81)
Smoking habit	1.58 (1.24, 2.02)*	Albumin	-2.71 (-3.65, -1.77)*
Unspecified comorbidity	4.70 (3.19, 6.91)*	Total bilirubin	1.01 (0.49, 1.53)*
Stroke	3.29 (0.85, 12.78)	AST	2.38 (1.73, 3.02)*
Kidney disease	3.16 (2.41, 4.15)*	ALT	0.40 (0.07, 0.74)*
CVD	3.11 (2.47, 3.91)*	Urea nitrogen	4.21 (2.66, 5.76)*
Hypertension	2.64 (2.17, 3.20)*	Creatinine	1.87 (1.24, 2.51)*
Malignancy	2.40 (1.77, 3.25)*	LDH	3.42 (2.49, 4.35)*
Diabetes	2.26 (1.88, 2.73)*	CRP	3.73 (3.02, 4.45)*
Pulmonary disease	2.05 (1.31, 3.21)*	Interleukin-6	3.27 (2.06, 4.49)*
Liver disease	1.65 (1.11, 2.46)*	ESR	0.17 (-0.31, 0.64)
		Procalcitonin	1.98 (1.21, 2.74)*
		Cardiac troponin-I	1.39 (0.70, 2.08)*
		Ferritin	2.00 (1.41, 2.60)*

* p-value <0.05

ALT: alanine transaminase; **APTT**: activated partial thromboplastin time, **AST**: aspartate transaminase; **BMI**: body mass index; **CRP**: C-reactive protein; **ESR**: erythrocyte sedimentation rate, **LDH**: lactate dehydrogenase.

[a] Excluded studies: Aloisio E et al., Asghar MS et al., Baqui P et al., Borobia AM et al., Chen F et al., Deng Y et al., Gavin W et al., Harmouch F et al., Krishnan S et al., Richardson S et al. and Yang X et al.
